# Designing for gender, age & proficiency: an empirical study of the cognitive ergonomics of dual-task interference in industrial human-robot collaboration

**DOI:** 10.3389/fnbot.2026.1222566

**Published:** 2026-09-15

**Authors:** Someshwar Roy, Igal Peretz, Lior Sayfeld, Yael Edan

**Affiliations:** 1 Intelligent Robotics Lab, Department of Industrial Engineering & Management, Ben-Gurion University of the Negev, Be’er Sheva, Israel; 2 AI and Emerging Technologies Lab, Connect-In Ltd., Glasgow, United Kingdom

**Keywords:** collaborative manufacturing, human-factors in Industry 4.0, human-robot interaction design, system design, UX research, cyber-physical systems, human-AI interaction design, Industry 5.0

## Abstract

**Introduction:**

This article reports an empirical study on Human–Robot Collaboration (HRC) under Dual-Task Interference (DTI). To the best of our knowledge, prior research has not investigated the intersection of Dual-Task Interference (DTI) and Human–Robot Collaboration (HRC) in industrial contexts. This study represents one of the first empirical attempts to understand DTI-induced human limitations in collaborative robotics, particularly in industrial HRC settings where stochastic interruptions and competing cognitive demands are common phenomena.

**Methods:**

The DTI phenomenon was implemented in a cognitive joint-assembly task involving 172 participants. Each participant collaborated with two robots while simultaneously performing a cognitive task designed to induce DTI during team-work. The performance measures included the number of Human–Robot interactions (measuring user engagement), total robot idle time (measuring team fluency), and the total number of completed cognitive tasks (measuring task performance).

**Results:**

Females exhibited significantly lower robot idle times (12%) and higher Human–Robot interactions (14%), indicating greater collaboration with the robot and more fluent team collaboration than males. Adolescents completed the cognitive task significantly faster than adults (21%) and experts (22%), whereas adults and experts demonstrated greater fluency in team collaboration. No significant differences were observed between novices and experts. Novices successfully bridged the skill gap by distributing their attention equivalently across all aspects of the team-work, resulting in performance comparable to that of experts.

**Discussion:**

The results reveal that (a) gender and age-group differences significantly influence HRC in DTI-based tasks, (b) performance speed in the primary task negatively affects the team fluency of HRC, and (c) effective team coordination skills are equally as important as task proficiency in HRC.

**Conclusion:**

This study contributes to design practice by demonstrating that collaborative robot learning algorithms can classify users into specific population clusters based on their characteristic patterns of collaboration with robots for improved performance and user experience (UX). Furthermore, task proficiency and team coordination emerge as two distinct and mutually independent skills in DTI-based tasks.

## Highlights

- Female participants were more collaborative with the robot and had more fluent team-collaboration than males.
- Adolescents were much swifter in their primary cognitive tasks with respect to adults and experts.
- Primary task performance speed negatively affects the team-fluency of H-R collaboration, suggesting an inherent trade-off between task performance and the cognitive costs of coordinating concurrent tasks.
- Good team co-ordination skill is equally important as task-proficiency in DTI-based HRC. A good team player can offset the edge of a well-acquainted expert.

## 1 Introduction

With the advent of Industry 5.0, the trend in industrial robotics is shifting away from robots enclosed in cages towards human-robot collaborative (HRC) systems (Baltrusch et al., 2022; Liu et al., 2022; Pizoń and Gola, 2023; Simões et al., 2022). Previous studies show that HRC systems provide improved efficiency and accuracy (Baltrusch et al., 2022) since they benefit from the strengths of each other while overcoming the limitations. But if human and robots share work- and time–space and a common goal, robots must become smarter, flexible and better aware of each other than they are today (Baltrusch et al., 2022; Liu et al., 2022). Cooperation between two humans is inherently intuitive (Wolf et al., 2018); however, collaboration between a human and robot introduces design challenges (Baltrusch et al., 2022; Gutman et al., 2021; Sanneman et al., 2021; Sheridan, 2020) that must be investigated in order to develop an efficient and user-friendly collaborative system.

Research in the area of human-factors and user-experience (UX) in human-robot collaboration is gaining significance (Baltrusch et al., 2022) as more and more small and medium sized enterprises (SME) and other less automated factories (e.g., semiconductor industry, aerospace) tend to introduce collaborative robots for tasks that were previously done solely by human (Choi et al., 2018; Sanneman et al., 2021). A collaborative robot is thus expected to be a multi-purpose plug-n-play robot that can collaborate efficiently with different types of populations (male or female, young or adult, expert or novice).

These collaborative robots are expected to assist human workers such that the tasks that require advanced perception and cognitive skills are done by humans and the repetitive or high precision tasks are done by the robots. This way, the overall task can be completed more efficiently and brings the need for active collaboration between the teammates throughout the product development cycle. Dual-task interference (DTI) is thus a common phenomenon in industrial H-R collaborative tasks (Fong et al., 2006). The phenomenon of dual-task interference (DTI) is, “a limitation of the human cognitive system in which the human brain must rapidly switch attention between multiple tasks that are being attempted at the same time” (Jenkins et al., 2016; Pashler, 1994). Thus, it is important to study its influence on different population groups for industrial human-robot collaborative tasks (Moniz and Krings, 2016).

Research in human-robot interaction (HRI) has been categorized into three dimensions (Mutlu et al., 2006): robot attributes (e.g., appearance and character), user’s personal factors (e.g., age, gender and mental models) and the nature of the task being performed (e.g., physical/cognitive; collaborative/competitive tasks). This study focuses on investigating the influence of user’s personal factors (gender, age group, proficiency) in performing a human-robot collaborative industrial task with dual-task interference. The populations examined in this study are adolescents versus adults, male versus female, and experts versus novices.

Previous studies revealed that employing an adaptive algorithm improves objective performance metrics in a human-robot collaborative task (Roy, 2016; Sayfeld et al., 2015). If the performance measures are, however, found to be population dependent, like, e.g., as in Heerink (2011), system designers can develop robot control strategies and algorithms that can identify and adapt more fluently to the needs of the user-group, e.g., as recommended by Baltrusch et al. (2022) in their design guidelines on HRC ergonomics and as implemented in the form of early explorations in Enayati et al. (2017), Realmuto et al. (2017), Skantze (2017), and Rossi et al. (2017). A recent study further strengthens this hypothesis. For example, Maroto-Gómez et al. (2022) has shown how adaptation in robot’s mode of interaction based on user-profiling into three groups (user—highly proactive, moderately proactive, not proactive) using a three step hierarchical decision making system has resulted in better user-experience and improved performance in HRC (Rossi et al., 2017); authors conclude that adaptation based on user-profiling resulted in “improving their (users’) likeability towards the robot and how intelligent they perceive the system”, thus, further reinforcing the need to investigate the ergonomics of designing population dependent human-robot collaborative systems.

The current research investigates the role of gender, age group & user-proficiency on human-robot (H-R) collaboration (HRC) with dual-task-interference (DTI) in industrial settings through a performance evaluation study. The DTI phenomenon was implemented in a cognitive joint-assembly task where a human collaborates with two robots and in parallel works on a cognitive task to accomplish the team-work. Prior research, as presented in detail in the next section on Scientific Background, has never attempted to combine DTI and HRC domain and highlights the need for investigation on human’s DTI-induced limitations in industrial HRC.

## 2 Scientific background

### 2.1 Role of human-factors in designing user-centered HRC system

One of the major HRC challenges with a human in the system control loop is the influence of human factors (Baltrusch et al., 2022; Charalambous et al., 2017; Maurice et al., 2017; Pearce et al., 2018) and cognitive factors (Biswas and Murray, 2016; Jenkins et al., 2016; Wright et al., 2018) and their implication for the design of human-robot collaborative systems.

Previous studies on performance evaluation in HRC showed how a human-aware robot can significantly improve team-coordination in collaborative tasks (Gutman et al., 2021; Lasota and Shah, 2015; Milliez et al., 2016; Oliff et al., 2020; Unhelkar et al., 2018) and how robot-human hand-over can be made seamless by considering human preferences (Huang et al., 2015; Roy and Edan, 2020). Temporal coordination in human-human collaboration is influenced by several intrinsic system variables (e.g., temporal preparation, perceptual latency, rhythm) (Bausenhart et al., 2010; Sanabria et al., 2011) and external factors (e.g., experience, fatigue, training, workload) (Lohani et al., 2017; Muhs et al., 2018) which has implications for HRC. Ergonomic studies on the influence of different human factors in HRC is thus critical (Baltrusch et al., 2022), e.g., as demonstrated in Heerink (2011), Maurice et al. (2017), Reinerman-Jones et al. (2017), and Wright et al. (2018) for designing user-centered HRC systems.

### 2.2 Dual-task interference phenomenon

The DTI phenomenon has been extensively investigated for different population groups and a variety of tasks (Batsakes and Fisk, 2000; Epling et al., 2018, 2019; Goddard et al., 1998; Hollman et al., 2011; Kimura and Matsuura, 2020; Korteling, 1991, 1993; Pilcher et al., 2016; Sit and Fisk, 1999; Wang et al., 2007; Woodham et al., 2016). In HCI literature, a previous study on DTI in personal ubiquitous computing show that dual tasks in the form of addressing smartphone notifications and instant messages consume significant cognitive resources and results in increased stress and decreased productivity (Jenkins et al., 2016). Study of DTI in driving and pedestrian crossing behavior is also very common (Strayer and Johnston, 2001; Tapiro et al., 2018).

Similar studies have been done in HRI, more specifically, in multiple robot-human collaboration to understand the influence of multiple robot control on perceived human workload (Adams, 2009; Crandall et al., 2005; Parasuraman et al., 2005; Prewett et al., 2010; Wong and Seet, 2017; Wright et al., 2018). While little differences were observed between one- and two-robot control tasks, the performance deteriorated significantly and human error spiked up as the number of robots that the human needed to attend to were increased to four or more (Prewett et al., 2010). The focus of these studies was to investigate the human workload, performance metrics, team performance and error rates while controlling multiple robots (UAVs, UGVs, robot-swarms) directly or remotely via teleoperation through web-interfaces.

While these studies shed light on the human control dimension suggesting that the human control of two robots in parallel balances the need for optimizing team performances and error rate beyond which the human performance deteriorates, the findings have little relevance to understand the influence of DTI on human-robot collaborative behavior in industrial context where the focus is on the fluent coordination between primary and secondary task in collaboration with one or multiple robots as a team. To the best of our knowledge, the study of industrial HRC for DTI-based tasks has not yet been investigated. This is a common scenario in assembly industries where a user must attend a primary and a secondary task in parallel. In DTI parlance, these primary and secondary tasks are the constant and the alternating demands of a task (Korteling, 1994). Such scenarios will become more common in the post-COVID era with the introduction of collaborative robots to automate tasks previously done solely by human.

### 2.3 Gender-based differences

Gender has always been perceived as an important factor in HRI and HCI due to the differences between men’s and women’s social behavior, socio-psychological processes and their social roles in the society (Eagly, 1987). The existing body of research on gender in human-robot interaction (HRI) can be classified into four groups (Nomura, 2017) namely, (i) the effects of gender characteristics in robotics design (robot gender, robot appearance and behaviors) (Carpenter et al., 2009; Niculescu et al., 2010) (ii) gender differences in interaction with robots (Chung-En, 2018; Kuo et al., 2009; Lin et al., 2012; Sandygulova and O’Hare, 2018) (iii) interaction effects between gender factors in different contexts and, (iv) the impact of the interaction between humans and robots with regard to situational factors, such as tasks and roles (Kuchenbrandt et al., 2014; Mutlu et al., 2006). The current research falls under the fourth group where the human gender differences in interaction with robots were studied under the influence of situational factors like, DTI, increasing cognitive work load and a time-bound industrial assembly environment.

Research on the inter-connection between task structure (competitive/cooperative) and gender revealed that the social experiences of female participants did not rely on the structure of the task whereas the experience of the male participants was more task dependent (Mutlu et al., 2006). Additionally, men showed greater desirability towards robots in cooperative tasks which indicated better user-acceptance (Mutlu et al., 2006). In another separate study, task performance in human-robot collaboration has also been shown to be influenced by the gender typicality of the given task (Kuchenbrandt et al., 2014).

Gender differences have also been observed in user acceptance of healthcare robots (Broadbent et al., 2009; Kuo et al., 2009). Khan (1998) explored users’ ideas, beliefs, perception (including robot appearance) and attitudes towards robotic assistance for several household tasks in domestic setting. The study showed that people were actually positive towards the idea of service robots, however, it found significant gender differences in the attitude towards the concept of robotic assistance for household chores. Integration of simple gender characteristics in robot designs has also shown to affect humans’ feelings and behaviors towards robots (Ghazali et al., 2018). This is of relevance to the robotics community because it has a direct effect on user-engagement and on the overall user-experience.

In HCI literature, gender differences were observed in e-learning acceptance (Ong and Lai, 2006; Wang et al., 2009), in the use of email (Gefen and Straub, 1997), in technology adoption decisions (Morris and Venkatesh, 2000) and also in self-efficacy and attitude towards computers (Busch, 1995). In literatures on behavior study, gender diversity is found to play an important role in human-human team collaboration and performance in workplace (Bear and Woolley, 2011). In social settings, gender differences were observed in subjective appraisals of collaboration and in cognitive collaboration of dyads (Margrett and Marsiske, 2002).

Most of the gender based HRI research focussed on the social context and on the subjective aspect of the interaction which has very little relevance to industrial robotics. Industrial HRC differs from social or domestic HRI due to time pressure, productivity constraints, safety requirements, and repetitive assembly demands. These factors reduce the relevance of subjective social metrics and emphasize coordination efficiency, workload tolerance, and fluency. Additionally, the industrial and manufacturing workplaces being mostly male-dominated professions, where women are likely to be in the significant minority, very little is known about the gender-based differences in the domain of HRC in industrial environments.

The need for investigation on gender-based differences is further motivated by a previous study that found female subjects were more productive than males in tasks involving assembly workstations (Garbie, 2014) and a recent study that found gender has an impact on workload capacity and task completion times and proposed “gender-adjusted models” for improving production line efficiency (Efe et al., 2018).

In the current study, the given task is cooperative in nature as in Mutlu et al. (2006) and the task required closer human-robot interaction as in Kuo et al. (2009), so a higher degree of user-engagement from men would be in consistence with the previous results. However, unlike previous studies, given the current experiment being set in a time-bound industrial assembly environment, females may exhibit better task performance as in Garbie (2014). Additionally, the current study requires the subject to work on a cognitive task in a DTI environment, so it is also equally probable that gender may not play any role at all as in Almajid and Keshner (2019). If however, gender is found to play an influencing role in DTI-based HRC, then such tasks could be accordingly allocated to the gender with higher efficiency and better coordination skills. Alternatively, if the collaborating robot is aware of the gender specific strengths and weaknesses in DTI-based tasks and hence their specific needs, then an adaptive algorithm could be designed accordingly to assist the collaborating partner in the way most useful and effective for the H-R team.

### 2.4 Age-related differences

The psycho-social development of individuals is generally divided into eight different stages starting from infancy to old age (Erikson, 1963): infancy (0–2 years), toddlerhood (2–4 years), early childhood (5–8 years), middle childhood (9–12 years), adolescence (13–19 years), early adulthood (20–39 years), middle adulthood (40–59 years), and late adulthood (60+ years). Age-related difference studies in HRI, HCI and DTI from three such age groups—adolescents (12–19 years), younger adults (university students, aged 21–29 years) and older population (>60 years) have been reviewed in this section.

In age-related differences literature in HRI, the studies generally focus on older population groups in comparison to younger adults. For example, Scopelliti et al. (2005) investigated the similarities and differences among three different age groups (young adults—mean age: 24.17 years; older adults—mean age: 53.5 years and elderly people—mean age: 75.88 years) in their attitude, preferences and perception for social robots. The older group was found to have more mistrust for new technologies, they responded with more negative emotions towards robots, and expected robots to be more serious than social with a minimum number of autonomous features like self-learning abilities. Differences in the perception of expressive behaviors of robots by elderly people and university students/young adults were reported in Chung-En (2018) and Nomura and Nakao (2010). Task performance and user-preferences in human-robot collaboration in the area of rehabilitation robotics also depended on the age group (Eizicovits et al., 2018; Feingold-Polak et al., 2018). In the context of industrial robotics, these age-group studies do not give enough information to model human behavior for industrial HRC systems.

Several studies in HCI, however, compared the task performance between adults and adolescents. Hourcade et al. (2004) studied how and why adults perform better than adolescents in a pointing task. Bilal and Kirby (2001, 2002) compared the performance of adolescents (7th grade students) and young adults (university graduates) in an online information seeking task and found influencing factors relevant for interaction design in HCI domain. Thus, there is evidence to suggest that examining age-related performance differences will broaden our understanding of human’s DTI induced limitations in HRC systems and its implications for developing similar interaction design guidelines in the area of industrial HRC.

Age-differences studies in the core DTI paradigm show that age differences have an important role in task performance in DTI tasks. Previous studies have consistently proved that younger adults perform significantly faster than older adults in DTI tasks (e.g., Batsakes and Fisk, 2000; Korteling, 1993; Kramer and Larish, 1996; Lorsbach and Simpson, 1988; McDowd and Craik, 1988; Salthouse et al., 1984; Somberg and Salthouse, 1982) and age differences meaningfully affected the perception of temporal demand and performance (Kang and Yoon, 2008). Another factor that may affect dual task performance is the relative complexity of dual tasks (McDowd, 1986). Older adults tend to be proportionally slower than younger adults as the complexity of the tasks being performed increases (McDowd et al., 1991).

These DTI studies, however, did not compare the performance of adolescents and young adults. While, it is clear that age-differences in adults affects DTI task performance, it is unknown if the same trend continues between adolescents and young adults and if an opposite trend might occur in the case of DTI in HRC similar to the pointing task in HCI (Hourcade et al., 2004). Furthermore, if adolescents are found to perform significantly better, their performance outcome can be used as a benchmark to understand the relative decline in performance curve over age.

### 2.5 Proficiency-based differences

User-proficiency based studies in HRI and HCI generally categorize the users into two groups—experts and novice. Experts are identified using different methods, each with its own set of advantages and disadvantages, as described in Shanteau et al. (2002). In medical robotics, an expert is generally trained through repeated trials until three consecutive near-optimal performance results are obtained (Labonte et al., 2010). In expert’s classification parlance, this methodology can be classified as a combination of *intra-person (within) reliability* and *creation of experts* through training (Shanteau et al., 2002). In the current experiment, *prior job-relevant experience* (Shanteau et al., 2002) was adopted as the methodology for classifying experts.

Proficiency-based differences have been widely investigated in the area of medical robotics (Chandra et al., 2010; Judkins et al., 2009; Kaplan et al., 2016) and tele-robotics (Bukchin et al., 2002; Enayati et al., 2017; McGinn et al., 2017) where the success of collaboration significantly depends on surgeon and operator skills, respectively. Factors affecting user performance are discussed in McGinn et al. (2017) and Meyer zu Borgsen et al. (2017). The outcomes of these user-proficiency studies, however, cannot be translated to develop HRC design guidelines for industries.

Research on user-proficiency in the domain of production and assembly systems has investigated how workers skill level and proficiency can impact the workflow, throughput and system productivity (Katiraee et al., 2019). Previous studies reveal that user-proficiency of factory workers can impact task processing time (Gong et al., 2018; Liu and Yang, 2019), production system cost (Rabbani et al., 2017), workload balancing (Sadeghi et al., 2018), smoothing (Xin et al., 2015) and safety (Katiraee et al., 2019). While these studies did not include proficiency-based differences in DTI environment, they show the significance of investigating user-proficiency for industrial HRC.

Proficiency based differences have also been investigated in the core DTI paradigm where existing literature have explored skill acquisition (Wang et al., 2007) and retention aspects (Batsakes and Fisk, 2000), and development of coordination strategies with changing pay offs (Wang et al., 2007; Sit and Fisk, 1999).

As more and more SMEs and other less automated factories (e.g., semiconductor industry, aerospace) tend to introduce collaborative robots for tasks that were previously done solely by human (Choi et al., 2018; Oliff et al., 2020), a foreseeable future scenario would be that the collaborating factory workers are proficient in their core primary tasks but new to the HRC and DTI environment (Moniz and Krings, 2016). From the current literature, it is unclear how that proficiency would influence their collaborative performance as a team in a DTI-based task. The proficiency-based differences examined in this study is novel in the sense that here the experience of the subjects is not in the DTI environment but on the primary task. Prior studies did not investigate the influence of primary task proficiency and its role in DTI environments. Primary task proficiency may take away the demanding cognitive load of this task from the experts and hence, it is hypothesized that it may give them an edge over novice to better manage the overall attention allocation, temporal and coordination demands of the DTI-based HRC system.

### 2.6 Collaborative task performance evaluation

Previous studies in the area of social robotics investigating age- and gender-based differences focused on subjective metrics and qualitative analyses for collaborative task performance evaluation (Kuo et al., 2009; Nomura and Nakao, 2010; Scopelliti et al., 2005). On the contrary, every significant DTI literature (Korteling, 1991, 1994; McDowd, 1986; Sit and Fisk, 1999; Strobach et al., 2012; Wang et al., 2007) including recent ones (Kimura and Matsuura, 2020; Rill et al., 2018) had only used the objective measures of performance. Furthermore, in a recent study, the strategic predictors of performance in DTI-based tasks are discussed (Rill et al., 2018), each of which are objective performance metrics.

Since, the current study was designed using the methodologies and common features of previous DTI-based studies (e.g., distribution of attention allocation through constant and randomized alternating demands, inclusion of pay-offs, making pay-offs discrete to allow subjects to strategize; elaborated later in Section 2), objective metrics were opted as the sole performance measures.

HRC system performance can be objectively measured using different methods such as operator workload and team performance (Adamides et al., 2017; Fong et al., 2006; Howard, 2007; Howard, 2005; Singer and Akin, 2011), human physiological responses (Sarkar, 2002), physical workload (Kim et al., 2015), engagement metrics (Anzalone et al., 2015), task metrics (Nikolaidis et al., 2015) and fluency metrics (Hoffman, 2019). The term “*fluency*” is defined as the level of coordination among the collaborating partners and measured using four common metrics—concurrent activity time, human idle time, robot idle time and functional delay—as proposed in Hoffman (2013, 2019) and used as system performance measures in Hoffman (2019), Lasota and Shah (2015), and Rahman (2018).

While there exists a body of research that shows the influence of DTI on an individual’s task performance in different domains (Kimura and Matsuura, 2020; Verhaeghen et al., 2003), very little is known about the effects of DTI on an individual’s team fluency. Existing studies did not investigate the paradigm of fluency in team-work under the influence of DTI which is an important aspect in the HRC domain.

### 2.7 Study overview and hypothesis

In this study, we aim to evaluate how team fluency is affected by DTI influence, temporal and collaborative task performance demands. This is done through an empirical performance evaluation of human-robot collaborative behavior under the influence of DTI. The study aims to understand if differences in gender, age-group, or user-proficiency impact a human’s ability to maintain team fluency while managing competing cognitive demands in a time-sensitive environment. Participants performed a primary cognitive task (solving a logic-based “Gravity Maze”) while simultaneously engaging in a secondary task (assembling and handing over Lego pieces to two robots). The experimental design is detailed in section 3.2 and 3.6 of the Methods section. The illustration in Figure 1 explains the Research Design detailed in section 3.9 in one snapshot.

**Figure 1:**
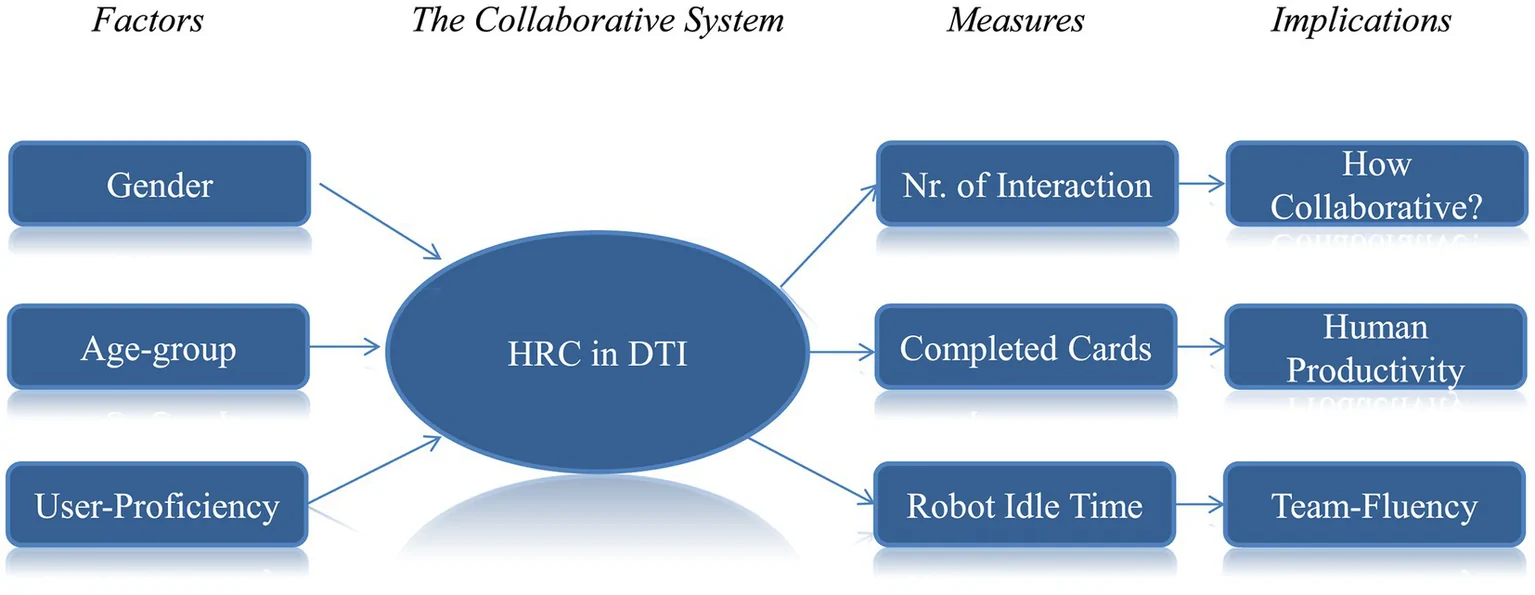
The research design.

The independent variables of this study, as mentioned in the very title of the paper is gender (male vs. female), age-group (adolescents vs. adults), and user-proficiency (experts vs. novices) while the dependent measures as detailed in section 3.5 on performance measures include the number of H-R interactions (measuring engagement), total robot idle-time (measuring team fluency), and number of completed cognitive tasks (measuring primary task performance).

Based on the existing literature in human-robot interaction (HRI), dual-task interference (DTI), and human factors in industrial engineering as discussed in section 2.1–2.6, the following hypotheses were tested:

H1 (gender-based differences): Given that females have been shown to be more productive in assembly workstation tasks, it is hypothesized that female participants will exhibit significantly higher team fluency (lower robot idle time) and greater user engagement (higher interaction counts) compared to male participants when performing HRC tasks under DTI.

H2 (age-related performance): Building on HCI evidence that younger populations outperform adults in cognitive pointing and information-seeking tasks, it is hypothesized that adolescents will demonstrate superior primary task performance (number of successful assemblies) compared to adults.

H3 (proficiency-induced cognitive load): It is hypothesized that experts, due to their prior job-relevant experience with the primary task, will possess a “cognitive edge” that allows for more efficient attention allocation. This reduced cognitive demand is expected to result in significantly better team-coordination and lower robot idle times compared to novice users.

H4 (task interference and fluency): It is hypothesized that higher performance speed in the primary cognitive task will negatively correlate with team-work fluency, as the cognitive cost of switching between tasks under DTI increases variability in H-R interactions.

## 3 Method

### 3.1 Participants

A total of 172 subjects (91 males, 81 females) took part in the experiment. The subjects include 137 undergraduate students aged 21–29 (70 males, 67 females), and 35 adolescents aged 13–14 (21 males, 14 females) from a youth project aiming to advance technology acceptance in children. In the adult population, there were 69 experts and 68 novices. To ensure the proficiency variable was not confounded by age, the mean age for the expert group (M = 23.4, SD = 1.8) and the novice group (M = 23.1, SD = 1.6) was kept statistically equivalent.

Experts were classified based on prior job-relevant experience as in Shanteau et al. (2002). The group of experts were recruited following their confirmation that (i) they are experienced in the primary task (have played and successfully completed the challenge of the Gravity Maze game, the primary task, multiple times in the past) and, (ii) familiar with the robot (had a minimum of 5 h of prior operational experience with the specific robot manipulators used through lab-based coursework in industrial engineering and automation). Importantly, while experts were proficient in individual tasks, they had no prior experience with the combined DTI environment, ensuring the interference effect remained a novel challenge for all groups. The undergraduate students received extra points as an incentive to participate in this study.

The goal of including adolescents as participants in this study was to use their performance as benchmark to understand how good or how bad the adults performed in the given collaborative task that required cognitive skills.

### 3.2 The robotic system

An H-R collaborative workspace was designed as part of this study and included two identical 5-degrees-of-freedom (DOF) revolute light-weight robot manipulators, a robot control system, the workbench and the collaborating human (Figure 2). The robots have a modular design powered by high-torque servo motors and controlled via a central microcontroller interface. Each robot was equipped with a two-finger parallel gripper optimized for picking and placing standardized Lego bricks. The total shared workspace was approximately 2.5 m^2^, with the robot bases positioned 60 cm apart to prevent any sort of mechanical interference while maintaining accessibility for the human collaborator.

**Figure 2:**
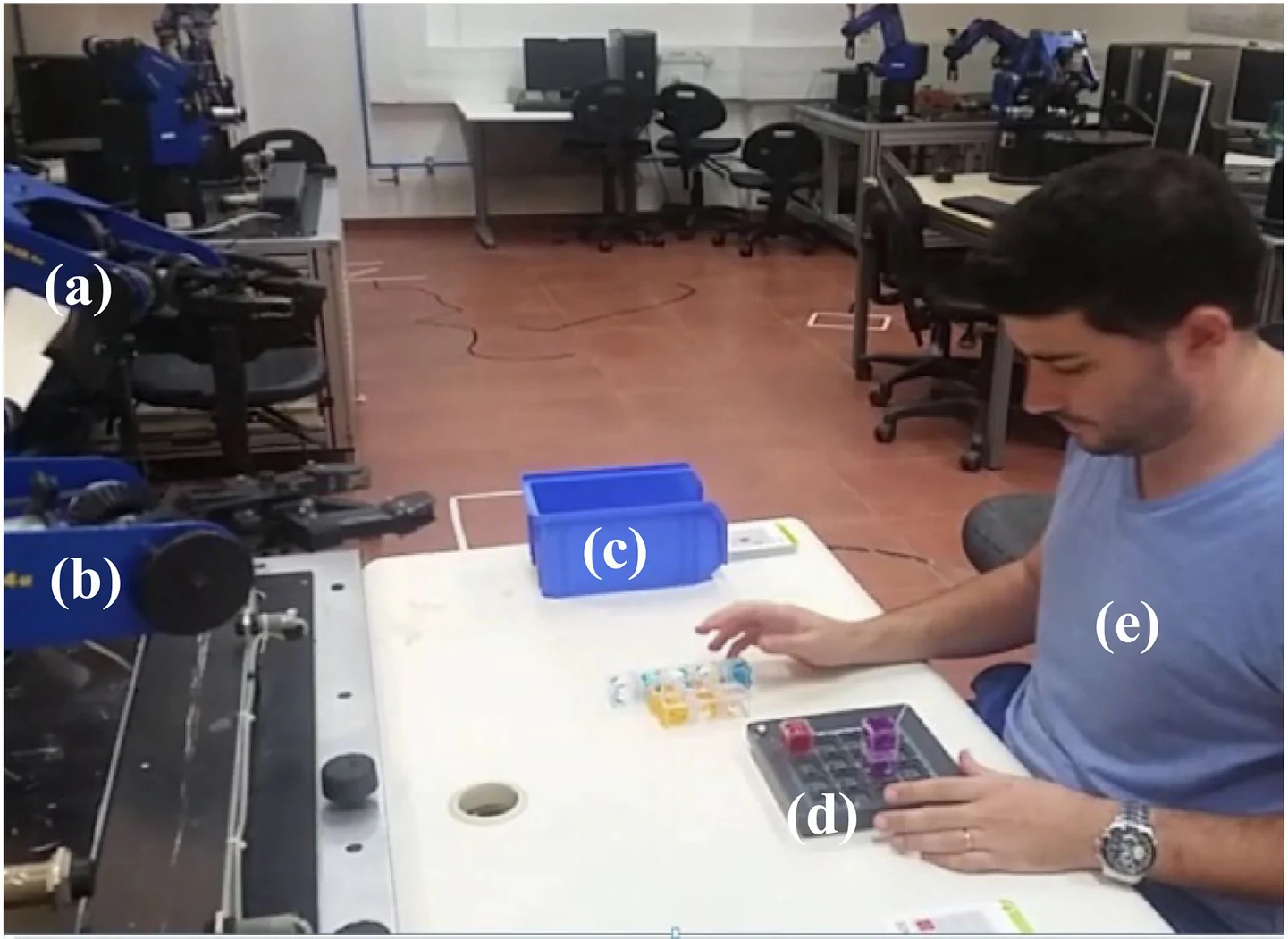
The H-R collaborative workstation: **(a)** the right robot, **(b)** the left robot, **(c)** the secondary task buffer (Lego pieces) on either side of the human, **(d)** the primary cognitive task, **(e)** the collaborating human.

The two robots were assigned with different assembly tasks and their operation was independent of each other. The robots, however, needed the finished assembly pieces (Figure 3c) from the human to continue with their respective assembly line tasks. This was when the HRC was required whereby the robot performs a pre-programmed trajectory to enter the shared workspace, collaborates with the human. The robot trajectories were developed using a standard Jacobian-based inverse kinematics solver to ensure smooth, predictable movements at a constant velocity of 0.1 m/s, ensuring safety and reducing human anxiety during close-proximity interaction.

**Figure 3:**
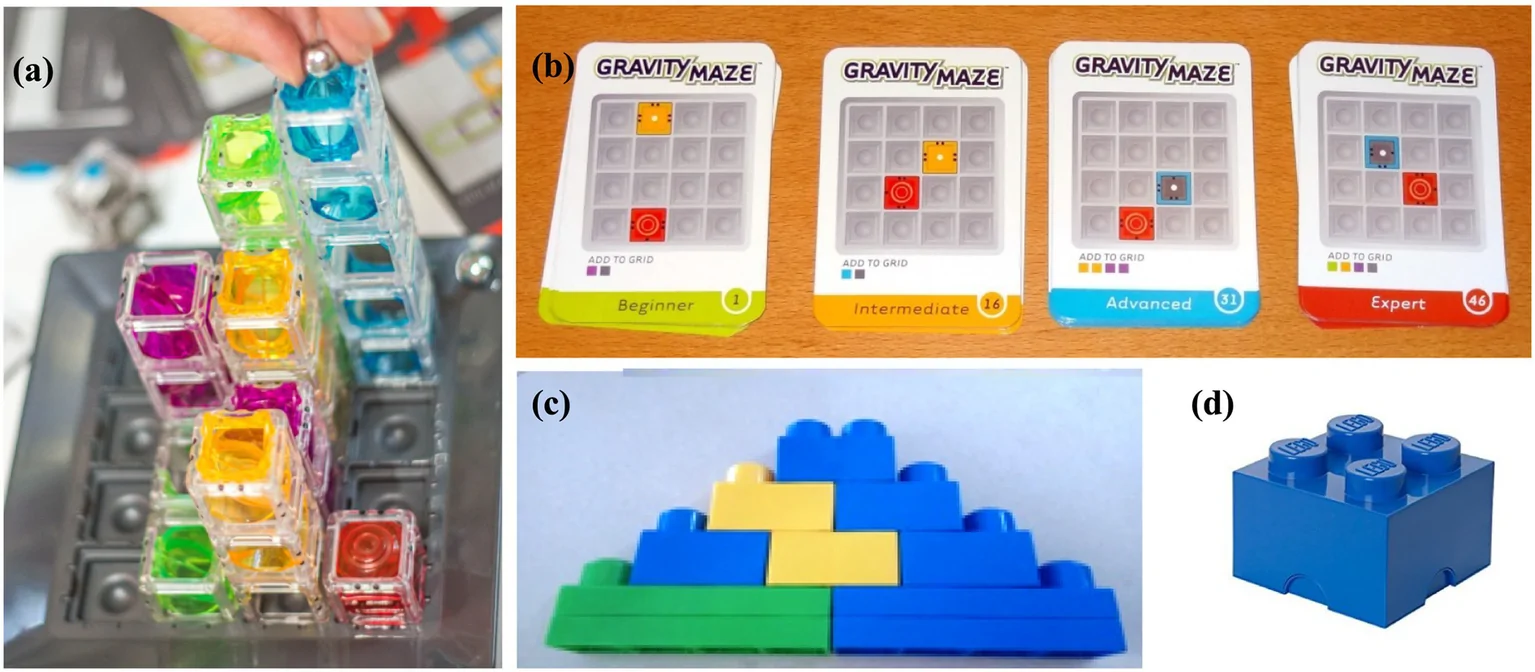
**(a)** The gravity maze cognitive game, **(b)** examples of the cards with increasing level of difficulty, **(c)** the secondary assembly task, **(d)** the Lego pieces.

### 3.3 The H-R collaborative scenario

The DTI phenomenon was implemented by splitting the human workload into primary and secondary tasks. The primary task included a cognitive assembly that required visual perception and problem-solving skills (Figure 3a). The human was required to solve a logical maze based on gravitational forces. Participants were presented with a card that defined the task (Figure 3b) and accordingly must build a path through the columns one constructed so as to move the ball into the red target destination. The task difficulty increased from card 1 to 5.

The aim of the primary task was to involve the human in a complicated industrial assembly that requires advanced perception and problem-solving skills (where human is better) in a time-bound environment. It is time-bounded because one of the three performance measures (elaborated in section 2.5) include the total number of successfully completed cards in the given timeframe.

The secondary task included assembling simple Lego pieces (Figure 3c) and handing them over to the respective robots as and when they needed so that they are able to continue with their respective tasks. The aim of the secondary task was to introduce an HRC team-work scenario where the human is seeking the assistance of robots for tasks where robots are better (e.g., routine automated assembly tasks).

The relationship between the primary cognitive task and the secondary robotic tasks is characterized by stochastic interruptions. While the human participant works continuously on the Gravity Maze (primary task), the robots operate on fixed 30 s and 60 s cycles, requiring assembly pieces (secondary task) to continue their operations. As shown in the experimental timeline (Figure 4), dual-task interference (DTI) peaks when both robots require assistance simultaneously, forcing the human to redistribute cognitive resources across three concurrent demands.

**Figure 4:**
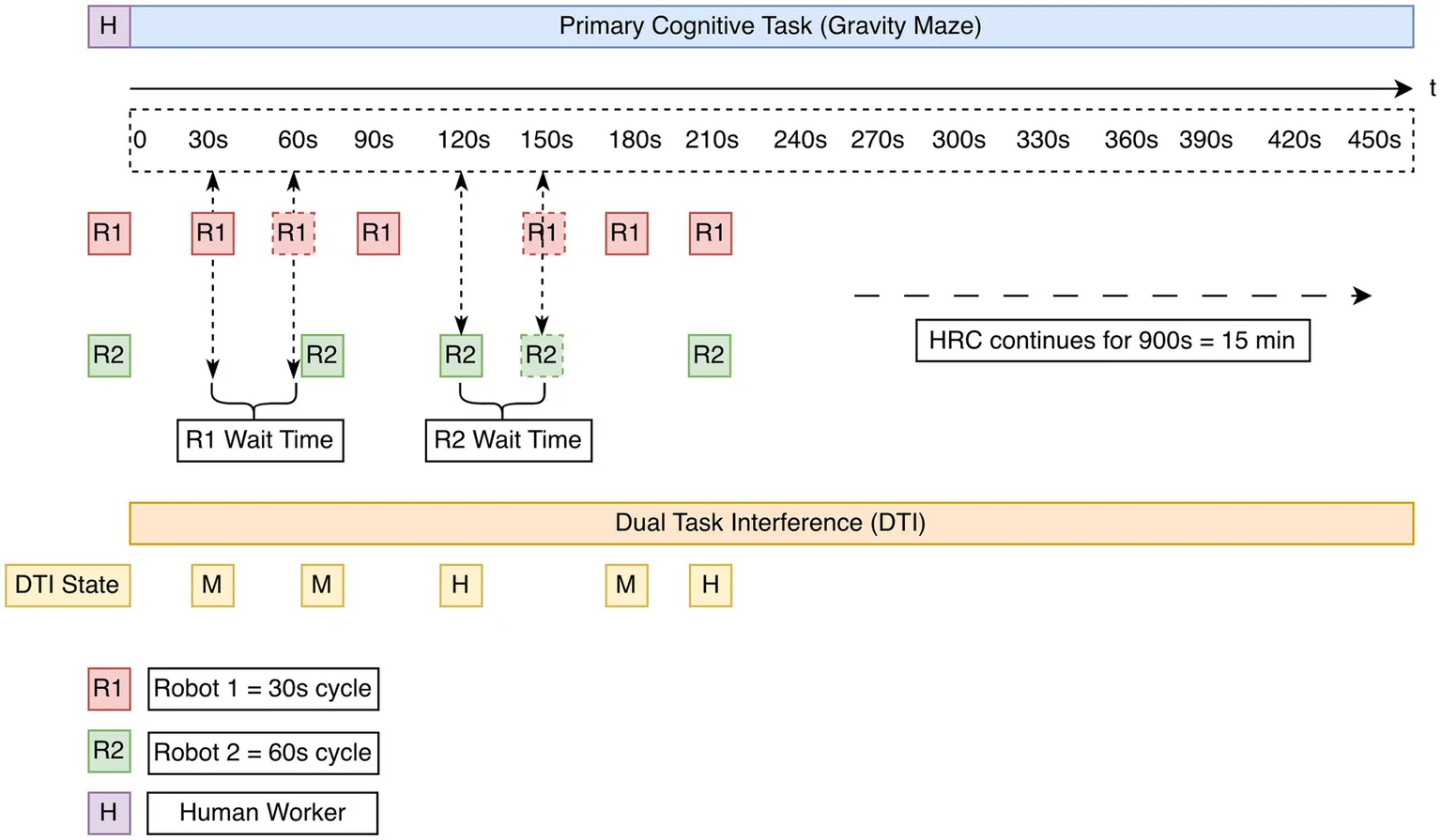
The experimental task timeline.

### 3.4 The control algorithm

The robot’s operational logic was governed by the timing-based control model for human-robot collaboration (Someshwar et al., 2012; Someshwar and Kerner, 2013) designed for synchronization with human workflows. The operational cycle times of the two robots were 30 s and 60 s respectively, the clock triggering right after a successful human-robot handover. These cycle times were set to create relatively fluent workflow and were pre-defined taking into account the operational demands and experimental design of the H-R collaborative scenario using a combination of heuristic analysis and empirical tests.

### 3.5 H-R coordination protocol

The coordination protocol is the pre-defined and explicit “rules of collaboration” between the partners in a mutually dependent task (Roy, 2016). In the experiment, the coordination protocol was “human never waits, the robot may need to wait.” The human never had to wait for the robot but continued with the primary task. The robots waited for the human to handover the necessary Lego pieces in every operational cycle to continue with its assembly task. With this H-R coordination protocol in place, the robot’s operational cycle is defined by five distinct functional states:

*a) Idle state*: The robot waits outside the shared workspace until its pre-programmed cycle time (30 s for the right robot; 60 s for the left robot) is reached.
*b) Transition state*: The robot enters the shared human-robot workspace to reach the point-of-handover (p-o-h).
*c) Waiting state*: The robot remains stationary at the p-o-h, waiting for the human to provide the necessary Lego assembly. The duration spent in this state constitutes the robot idle time used in performance metrics.
*d) Handover state*: Upon detection of the assembly piece, the robot executes a pick-and-place movement to acquire the part from the human.
*e) Task completion*: The robot transports the piece to the designated assembly line area and then returns to the idle state to reset the cycle.

### 3.6 The performance measures

The performance measures included the number of interactions between the human and the robots, the total robot idle time and the total number of successfully completed cards/cognitive task. The total number of H-R interactions measured the user-engagement of the human with the robot and the team-work. It measured how collaborative the human was with the robot in a time-bound environment while balancing the needs of one’s primary workload and team-objective. A higher number of H-R interactions indicated higher user-engagement and better suitability for H-R collaborative tasks.

The total robot idle time is the sum of the idle times of both the robots during the entire period of their operation. It was indicative of the collaborative work efficiency and team-fluency. A higher number of H-R interactions with minimal robot idle time are indicative of a good team-player in HRC tasks.

The total number of successfully completed cards/tasks in the given time was the third performance measure. It was indicative of the human performance in cognitive workload. The human assembly time of each card was also recorded. The assembly times of each card were indicative of the learning curve and acquaintance with the primary task. The first task (the first card) was intentionally kept relatively simple and did not require special cognitive skills and was specifically indicative of rapid learning of the goal and understanding of the job.

### 3.7 Experimental design

An industrial H-R collaborative scenario was designed using the system described in Section 2.1 inside the Intelligent Robotics Lab at Ben-Gurion University of the Negev (Figure 2).

The two robot manipulators were placed at the two corners of the workbench on one end and the human workspace is on the opposite end. The two robots were referred to as the left robot (LR) and the right robot (RR) for ease of understanding of the subjects. A chair was provided for the subjects to ensure H-R collaboration in sitting posture. This was done to recreate scenarios of longer sessions of HRC where the human may need to collaborate in a sitting posture (e.g., in the semiconductor industry). The primary task of the human was placed at the centre of the human workspace to indicate its primary nature. The Lego pieces were provided in a blue container on the right hand side for the secondary task.

The cycle time of the RR and LR were set as 30 s and 60 s to keep the minimum and maximum time intervals between two robotic interventions as 0 s and 30 s, respectively. These values were derived using a combination of heuristic analysis and empirical tests and, with the goal to design an experimental scenario where (i) the intervention is random and relatively unpredictable for the human (ii) the intervention load (DTI) varies randomly during the course of the entire experimental session (iii) avg. human/robot waiting time is not too high.

As the experiment progresses, the intervention becomes more random and unpredictable since it depends upon the interplay of robot waiting time, human waiting time in the preceding and the current H-R interaction cycles together with the increasing cognitive complexity of the primary task which would demand attention allocation away from the interventions. While the intervention time vary between the two pre-defined limits (0 s and 30 s) in every interaction cycle, it also influences the interference load. The shorter the intervention time between LR and RR, higher is the intervention load (DTI) of the collaborating human.

The timing-based control model for H-R collaboration, as used in this study, demanded the human to adapt oneself accordingly and balance the needs of the primary task and the team-objective instead of the robot adapting to the increasing cognitive workload of the human with time. This ensured the success in this joint-task was dependent on the collaboration skills of the human. The experiment was intentionally designed in this way to investigate the collaboration and DTI skills of the different populations.

In summary, the experimental design synthesized the primary and secondary tasks into a continuous 15-min session to investigate human performance under stochastic demand. As illustrated in the experimental task timeline (Figure 4), the collaborative workflow followed a non-sequential, interrupt-driven pattern. While participants worked continuously on the primary cognitive task, the robots operated on independent cycles of 30 s and 60 s. These cycles triggered “intervention windows” where the participant had to pivot to the secondary assembly task. Because the cognitive complexity of the maze varied, the interference load was unpredictable, peaking during “Double Interventions” when both robots required assistance simultaneously. This specific timing, combined with the time-acceleration penalties described in the following section, ensured that success was dependent on the participant’s ability to balance primary task speed with team-work fluency (see Figures 5, 6).

**Figure 5:**
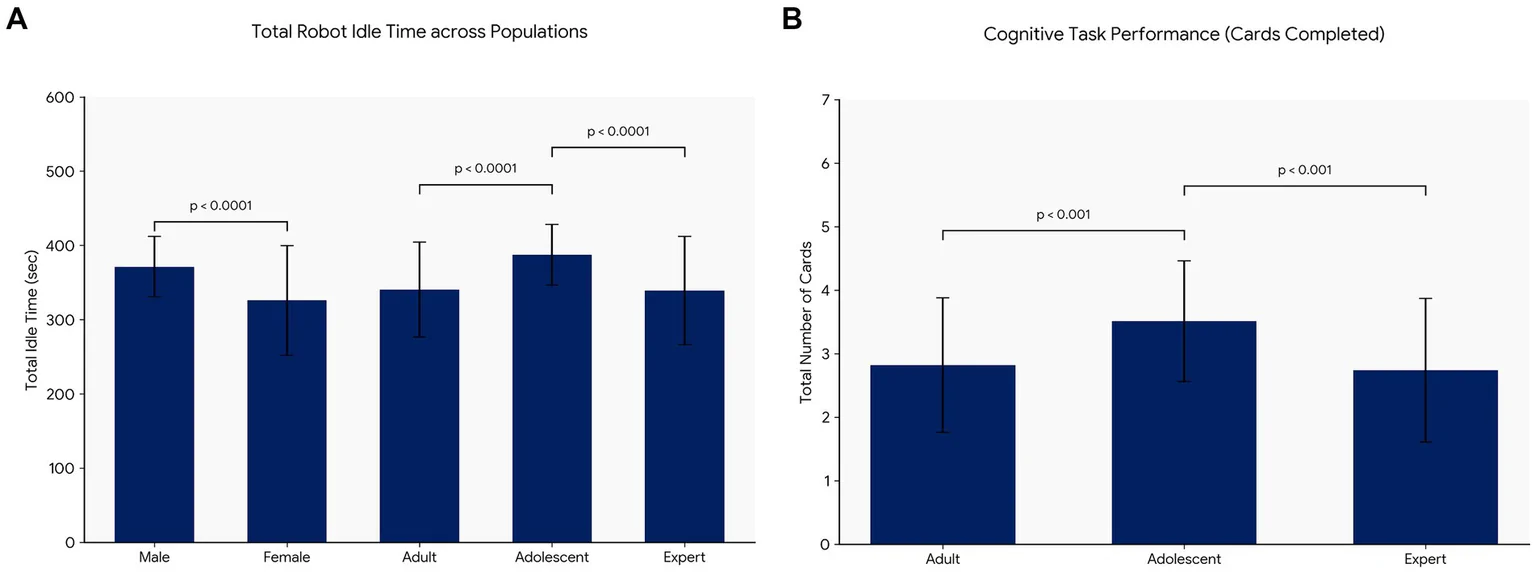
**(A)** This graph compares the team fluency (measured in sec) across the significant independent variable groups; females and adults/experts show significantly lower idle times, indicating higher team-work fluency compared to males and adolescents, respectively. **(B)** This graph compares user-engagement based on the frequency of interactions. Female participants exhibited a significantly higher number of interactions compared to males, indicating greater engagement with the robotic partners.

**Figure 6:**
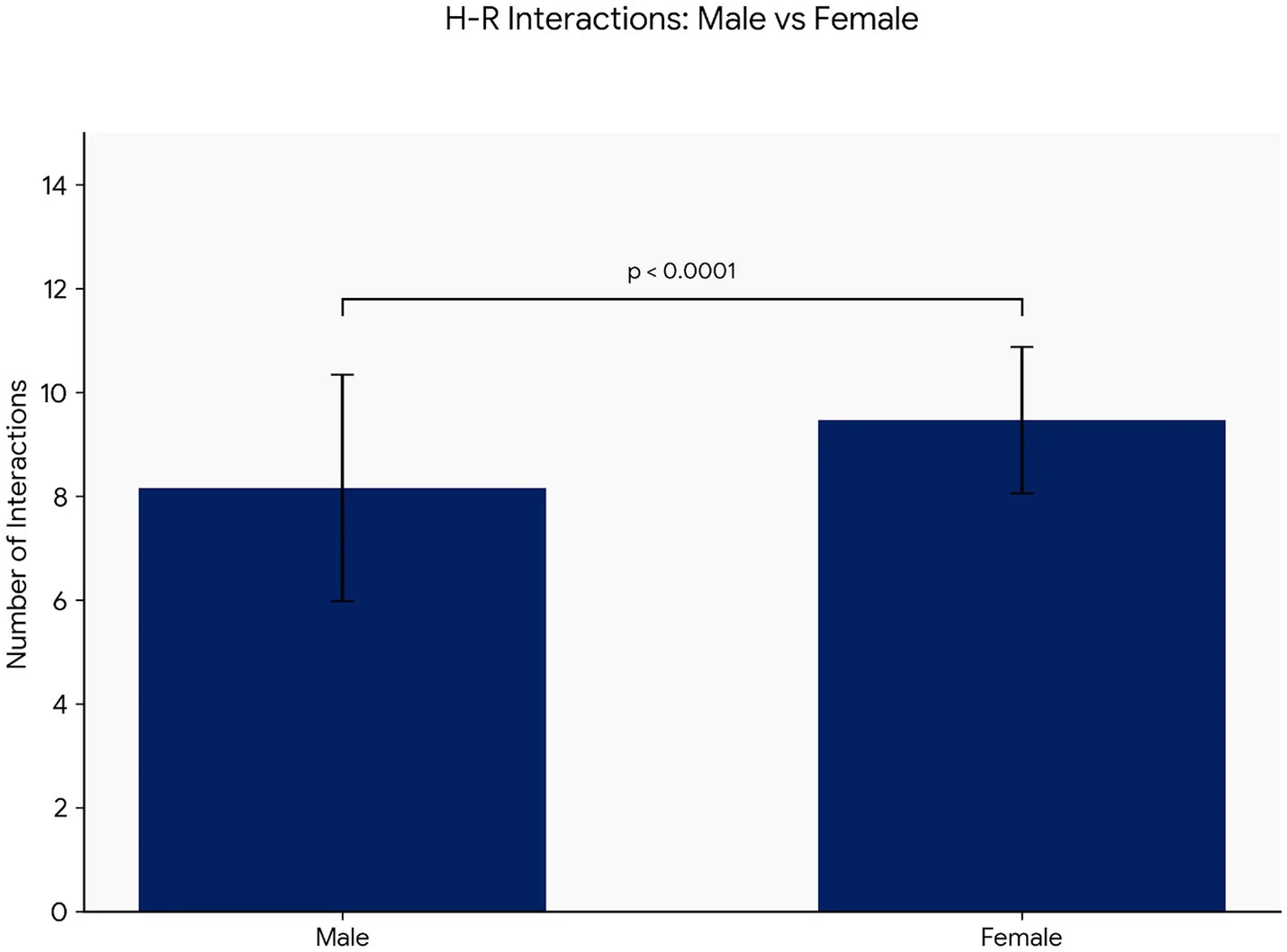
This graph compares the primary task performance through the number of successfully completed cards. Adolescents significantly outperformed both adults and experts in primary task completion, despite having lower team-work fluency (as seen in Figure 5A).

### 3.8 Experimental conditions

The duration of the actual experiment was 15 min during which all the subjects performed the same task with the same experimental conditions. No specific time constraint was imposed for each card since these tasks were cognitive in nature. While the subjects were asked to solve as many cards (primary task) as possible, they were noted that:

a) The clock would run 2× faster whenever the left robot was waiting for the human.
b) The clock would run 3× faster whenever the right robot was waiting for the human.
c) The clock would run 6× faster whenever both the robots were waiting for the human.

These penalties were set to motivate the subject to distribute one’s attention equivalently on the primary and the secondary task, to collaborate actively with the robot and to emulate the importance of fluent team-work. They were also representative of the cost of robot idle time on the team-work. The 3× penalty of the right robot is indicative of its higher task priority over the left robot’s task in the designed production environment. Adding a pay-off or a cost component to the task environment is a common feature in DTI-based studies and the pay-offs need to be explicit (Batsakes and Fisk, 2000; Wang et al., 2007; Farmer et al., 2018) to allow subjects to strategize accordingly. Both of these DTI characteristics have been implemented in the current study. All data collection was automated through the integrated sensor system.

### 3.9 Experimental procedure

Subjects received experimental instructions in written and verbal form. Subjects were allowed to use their watch. However, they were not given any information on the amount of time they lost due to overclocking when the robot was idle. No break session was provided during the course of the task. No subjective measures were used in this experiment. This research complied with the tenets of the Declaration of Helsinki and was approved by the University’s Human Subject Research Committee. Informed consent was obtained from each participant.

### 3.10 The research design

The research design included a Human-Robot collaborative system for DTI-based tasks, denoted in short as, *HRC in DTI* (Figure 1).

The factors being investigated are *gender*, *age-group* and *user-proficiency*, while the performance measures are *number of interactions*, *cards completed* and *robot idle time* with their corresponding implications noted on the far right.

The current experimental design utilized gender, age and user-proficiency as naturalistic proxies for cognitive readiness and task-switching capability and therefore individual differences in cognitive abilities have been excluded. This aligns with the primary focus of the research to investigate the *interaction* between established population demographics and the ergonomics of HRC in a DTI environment, rather than isolating the underlying neurocognitive mechanics.

## 4 Results and discussion

### 4.1 Analyses

The independent variables were gender, age group, and user-proficiency with the three performance measures defined as dependent variables. A 2-way ANOVA analysis was conducted between—proficiency & gender [2 (proficiency type: experts, novice) × 2 (gender: adult male, adult female)] and age group & gender [2 (age group: adolescents, adults) × 2 (gender: male, female)].

Since proficiency levels were only assigned to adults, the proficiency & age group interaction was excluded from this analysis and in the proficiency & gender interaction, only adult population was analyzed. When the interaction was found to be significant, a one-way ANOVA analysis was subsequently conducted to check the main effects.

Interaction effects were found between age group and gender for total idle time [*F*(1,168) = 5.9, *p* < 0.016, *η*_p_^2^ = 0.034] and number of cards completed [*F*(1,168) = 4.0, *p* < 0.04, *η*_p_^2^ = 0.024]. No significant interaction effects were found between proficiency and gender. While, the proficiency & age group interaction study was not possible, a one-way ANOVA analysis was done between experts and adolescents. All results with significant differences between populations (*p* < 0.001) are presented in Table 1 and discussed below.

**Table 1:** Results of the experimental study which were statistically significant.

Performance measures	I.V.	Mean	S.D.	*F*	Sig.	Conclusion
	Male and female					
Total idle time (sec)	M	371.38	40.51	25.78	0.0001	F > M
	F	325.94	73.8			
Nr. of interactions	M	8.16	2.18	21.05	0.0001	F > M
	F	9.47	1.41			
	Adult and adolescents					
Total idle time (sec)	A	340.46	63.84	16.99	0.0001	A > C
	C	387.26	40.83			
Nr. of cards	A	2.82	1.06	12.42	0.001	C > A
	C	3.51	0.95			
	Adolescents and experts					
Total idle time (sec)	E	339.17	72.89	13.1	0.0001	E > C
	C	387.26	40.83			
Nr. of cards	E	2.74	1.13	12.05	0.001	C > E
	C	3.51	0.95			

M/F, male/female; A/C, adult/child; E, expert; “>” indicates better performance; IV, independent variable.

### 4.2 Gender-based differences: results and implications

Females had significantly more interactions with the robot [14% more, *F*(1,170) = 21.05, *p* < 0.0001, *η*_p_^2^ = 0.110] and had lower robot idle times [12% lower, *F*(1,170) = 25.78, *p* < 0.0001, *η*_p_^2^ = 0.132] than the males. Females also had the highest number of interactions with the robot among all the population groups (Figure 1). However, there was no significant difference between the gender groups in the total number of cards completed. This indicates three characteristics: (a) females distributed their attention equivalently towards both the primary and the secondary task while still being equally efficient as males in the primary task (b) females worked more collaboratively with the robot to achieve the overall goals (c) they valued team-fluency and the cost of a waiting team-partner.

Previous research in social robotics indicates that men typically exhibit higher engagement with robots in cooperative tasks and those involving close-proximity interaction (Mutlu et al., 2006; Kuo et al., 2009). Conversely, recent findings in the core DTI paradigm suggests that gender may not significantly influence performance in DTI-based cognitive tasks (Almajid and Keshner, 2019). The current study integrated these elements by designing an experiment involving a cooperative, close-proximity task within a DTI-influenced industrial environment. Our results provide new evidence favoring female performance in these settings. This insight is particularly significant as prior robotics research often relied on subjective evaluations, whereas existing DTI studies were conducted outside of industrial contexts.

In terms of the objective metrics in the current study, female participants were more collaborative with the robot. Additionally, the team collaboration was also far more fluent since they valued the cost of a waiting team-partner more than males. This supports previous findings in the domain of industrial manufacturing research that female subjects were more productive than males in tasks involving assembly workstations (Garbie, 2014) and that gender has an impact on workload capacity and task completion times (Efe et al., 2018). The outcome of this study further stresses the need for “*gender-adjusted models*” in factories as proposed in Efe et al. (2018) for improving production line efficiency.

### 4.3 Age-based differences: results and implications

Adolescents performed their primary tasks significantly faster than adults right from the beginning and consequently accomplished 20% higher [*F*(1,170) = 12.42, *p* < 0.001, *η*_p_^2^ = 0.068] number of successful assemblies (cards). Furthermore, *adolescents were even faster than experts* and successfully completed 22% higher [*F*(1,102) = 12.05, *p* < 0.001, *η*_p_^2^ = 0.106] number of successful assemblies. There was no significant difference in the number of H-R interactions among the two age-groups, however, the team collaboration was significantly more fluent for adults and experts as compared to adolescents with 12% [*F*(1,170) = 16.99, *p* < 0.0001, *η*_p_^2^ = 0.091] and 12.5% [*F*(1,102) = 13.1, *p* < 0.0001, *η*_p_^2^ = 0.114] lower robot idle times, respectively.

These results suggest that the adolescent group possessed superior problem-solving efficiency and higher fact/recall processes within the context of the cognitive-intensive primary task which translated into three characteristic patterns of this group: (a) adolescents’ learning rate was higher than that of the adults (b) adolescents were swifter in their primary tasks and better managed the demands of DTI-based tasks (c) higher primary task throughput inducing a “momentum” negatively affected the fluency of H-R collaboration.

While previous literature suggests a link between cognitive performance and digital games engagement in younger digital-native populations (Chuang and Chen, 2009; Kovess-Masfety et al., 2016), this study did not specifically measure the participants’ prior video game experience. Consequently, the superior task completion rates observed in adolescents are treated as a behavioural benchmark for cognitive-intensive DTI tasks, rather than being attributed to specific external habits. Despite this primary task advantage, adolescents exhibited higher robot idle times, suggesting that their intense focus on primary task momentum negatively impacted the temporal coordination and fluency of the H-R team.

Since there exist no significant difference between the groups in the total number of interactions, we find that the engagement of adolescents and adults with both the robots were similar. This supports the findings of some recent studies in rehabilitation robotics comparing the experience of older and younger age groups in interacting with the robot against a non-embodied system where engagement of both the population groups with the robot were found to be similar (Eizicovits et al., 2018; Feingold-Polak et al., 2018).

From DTI-perspective, adolescents managed to deliver the same level of user-engagement with the robots as the adults but coupled with higher performance in primary tasks. This indicates that adolescents were able to perform and manage the demands of primary and secondary tasks better than adults. It supports previous findings that age-differences has an important role in task performance in DTI-based tasks (Kang and Yoon, 2008; Korteling, 1993; Kramer and Larish, 1996). In this study, the complexity of the primary task increased with every new card and temporal costs kicked in with robot idle times in the successive H-R interaction cycles thereby adding more complexity to the overall task. Adolescents performing better than young adults further supports previous findings that older adults tend to be proportionally slower than younger adults with increasing task complexity (Sit and Fisk, 1999; Verhaeghen et al., 2003). While the decline in performance in older adults in previous studies is due to their diminishing cognitive skills, in this case, higher fact/recall processes and better problem-solving skills probably helped adolescents better handle the given task complexity. Having said that, literature in age-related DTI-based studies show that there is little agreement among researchers as to the mechanisms underlying these performance differences (Batsakes and Fisk, 2000; Lorsbach and Simpson, 1988; McDowd and Craik, 1988; Salthouse et al., 1984; Somberg and Salthouse, 1982).

Previous studies, however, did not compare the performance of adolescents and young adults. This study further builds on previous works by concluding that the same trend continues between adolescents and young adults. Additionally, the performance outcome of adolescents in this study can now be used as a benchmark in future research to understand the relative decline in performance curve over age in DTI-based tasks.

Furthermore, previous studies never investigated the concept of fluency in team-work done under the influence of DTI. This is an important aspect in the domain of HRC. The adolescents being too focused on their primary cognitive task, each H-R interaction meant a break in their momentum and came with a temporal and cognitive cost of switching between tasks (Rogers and Monsell, 1995; Yeung and Monsell, 2003). This probably is the reason for higher robot idle times which resulted in higher variability in H-R interaction and lacked the fluency in their team-work with the robots. This implies that higher performance speed in the primary task done under the influence of DTI came at the cost of negatively affecting the fluency of H-R collaboration. It supports the findings of a previous study (Roy and Edan, 2017, 2020) done on human-human (H-H) collaboration that shows there exist higher variability in H-H handover for “high-yield” teams (higher throughput) while the “low-yield” teams, being slower in their performance and thus resulting in low-yield, had better team-coordination following the principle of Fitt’s law.

The results of the current study give a new insight on the influence of DTI on an individual’s temporal coordination skills, about which very little is known today. Few studies investigated the inter-task coordination aspects of DTI (Liepelt et al., 2011; Strobach et al., 2012; Wolff and Cohen, 1980) which shed very little or no information on the temporal aspects of coordination.

### 4.4 Proficiency-based differences: results and implications

There is no significant difference in the total number of successful assemblies between experts and novices [*F*(1,133) = 0.696, *p* < 0.406, *η*_p_^2^ = 0.005]. Also, there is no significant difference between the population in robot idle time [*F*(1,133) = 0.005, *p* < 0.942, *η*_p_^2^ = 0.000] and the total number of interactions [*F*(1,133) = 1.992, *p* < 0.160, *η*_p_^2^ = 0.015]. Expert’s proficiency in the primary task and familiarity with the robot did not provide them with an edge in their performance in the DTI environment to which they were unfamiliar. The DTI environment acted as a kind of “equalizer” for experts and novices.

Results indicate two characteristics: (a) the robot idle time difference between the populations being insignificant, both novices and experts valued the cost of a waiting partner and maintained equal fluency in H-R collaboration (b) unfamiliarity with the robot and with the primary task does not imply inefficient or slower team performance.

While experts held an advantage in primary task familiarity, novices successfully matched their output by adopting a more balanced attention distribution strategy. By allocating equivalent focus to both the primary cognitive task and the secondary robot interventions, novices effectively minimized the cumulative time penalties that would otherwise have widened the performance gap. This suggesting that in DTI-based HRC, *strategic coordination*, rather than solo task-proficiency, is the primary driver of success.

This study reveals a new insight that primary task proficiency does not play any role in DTI task proficiency. DTI skills can, however, be acquired, retained and transferred across other tasks (Batsakes and Fisk, 2000; Liepelt et al., 2011; Wang et al., 2007). Thus, in order to develop an efficient workforce with skills in industrial HRC, strategic training of factory workers is critical (Michaelis et al., 2020). Training modules on DTI-based activities should be offered that improve their overall coordination strategies and make them better prepared to handle distributed attention allocation and temporal demands of the system. Industrial trainings that are specifically targeted towards the primary task are of little relevance in the DTI-based HRC. Previous studies also show that performance gains acquired through trainings are better retained by younger adults as compared to older population (Batsakes and Fisk, 2000). So, a better strategy would be to use both skill retention and performance gain as a metric to evaluate training outcomes for allocating industrial workforce in DTI-based HRC.

### 4.5 Correlations among performance measures

To understand the dependence between the performance measures, Pearson’s correlational analyses were conducted (Table 2). Results indicate there is a strong negative correlation (*p* < 0.0001) between the total number of interactions and robot idle time. As the number of interactions increases, the total idle time decreases.

**Table 2:** Correlations among performance measures.

Performance measures	Total idle time	Sig.
Nr. of cards	−0.28	0.0001
Nr. of interactions	−0.344	0.0001

This indicates that subjects who were extra careful about the robot idle time and its importance in maintaining team-fluency were more collaborative with the robot and were more successful in accomplishing the team-objective. The female subjects are the best example of this conclusion. They had lower idle times and higher number of interactions and were better collaborative partners than the males.

The other result indicates that there is a strong negative correlation (*p* < 0.0001) between the total number of cards completed and the total idle time. This is because, as the robot goes idle (waits for the human), the clock starts ticking faster than usual and hence, the time available to the human decreases from the usual 15 min. This results in accomplishing lower number of primary tasks (cards).

## 5 Conclusion

Results reveal that gender and age group differences significantly influence the objective performance in H-R collaboration in DTI-based tasks. Given the lack of data investigating at the intersection of DTI and industrial HRC, this is the first study of its kind that attempts to combine the fields of DTI and HRC and highlights the need for further investigation and awareness about human’s DTI induced limitations within the Industrial Robotics community. The implication of this study for industrial practitioners and designers have been summarized below.

### 5.1 Contributions to design practice

*i) Designing adaptive algorithms via user-profiling*:

Collaborative robot algorithms can be designed to classify human partners into specific clusters based on gender, age group, and identified characteristic patterns of interaction. By recognizing these demographic and behavioural markers, systems can adapt their control strategies in real-time to improve overall team performance and enhance user experience.

*ii) The four-tier user-proficiency model*:

Solo task-proficiency and team-coordination are distinct and mutually independent skills in DTI-based environments. This study demonstrates that high performance in a primary task does not inherently translate to effective team-work fluency. This independence leads to four specific user-proficiency models based on the intersection of primary task expertise and team-coordination quality: novice/expert in the given primary task and good/poor team-player and vice-versa (Table 3). This four-tier categorization of user-proficiency, defined by the intersection of primary task expertise and team-coordination skills, finds direct empirical support in the behavioural patterns observed across our study groups.

- Novice/Good team-player: Exemplified by the novice cohort in this study, who bridged the task-familiarity gap by prioritizing high engagement and attention distribution to minimize robot idle time.
- Expert/Poor team-player: Reflected in the performance of adolescents and certain experts; while these participants achieved superior primary task throughput, their focus on maintaining task momentum resulted in higher robot idle times and reduced team-work fluency.
- Expert/Good team-player: Characterized by high primary task throughput coupled with minimal robot idle time.
- Novice/Poor team-player: Characterized by both low primary task throughput and poor team coordination.

**Table 3:** User-proficiency models.

Solo-task proficiency	Team-coordination skills	User-proficiency models	Relevant cluster from this study
Expert	Good	Expert in solo task & good team-player	Individual-specific case
Expert	Poor	Expert in solo task but poor team-player	Adolescents & experts
Novice	Good	Novice in solo task but good team-player	Novices
Novice	Poor	Novice in solo task & poor team-player	Individual-specific case

A collaborative robot capable of classifying a human partner into one of these four clusters can optimize its interaction strategy to balance productivity with team fluency.

*iii) Monitoring and mutual adaptation*: Design practitioners must account for the negative impact that high performance speed in a primary task can have on team productivity due to the cognitive cost of task-switching. Systems should ideally monitor performance speeds to ensure that primary task efficiency does not hamper team fluency. Implementing visual or auditory notifications can alert the human to adjust their pace to an optimum level, facilitating human-robot mutual adaptation (Nikolaidis et al., 2017a; Nikolaidis et al., 2017b).
*iv) Workforce training for DTI environments*: Efficiency in HRC can be improved by developing DTI-specific skills in the workforce. As noted previously, DTI task proficiency can be acquired, retained, and transferred across other tasks, practitioners should develop training modules specifically for factories introducing HRC in the floor. These modules should focus equally on performance gains and skill retention metrics to effectively prepare workers for the distributed attention demands of collaborative systems.

### 5.2 Limitations and scope for future work

The current study did not include subjective evaluation of the user-experience of H-R collaboration. Researcher interested in measuring the subjective outcome may use the standard User-Experience Questionnaire (UEQ or UEQ-S) (Schrepp et al., 2017). While the DTI phenomenon was implemented, its influence on overall team performance was not compared with single task conditions (with no secondary tasks or external interventions) like (e.g., Korteling, 1991, 1994).

Future research based on the findings of this article may look into the threshold limits of primary task performance speed in DTI-based HRC that provide an optimum balance between higher task performance, user-engagement and team fluency. It could be assessed if the decline in team-fluency with higher efficiency in the primary task is influenced by gender. It also brings into light a new question, whether the balance between team-fluency and team productivity in DTI-based tasks can be trained over repeated trials, e.g., as in Liepelt et al. (2011) and if so, can this performance gain be retained in general like (e.g., Batsakes and Fisk, 2000; Liepelt et al., 2011) or is this case-specific. Another interesting aspect to investigate is if the acquisition of team-fluency skills is transferrable with changing tasks and task demands or is it task- and case-specific. Performance evaluation studies should be performed over a longer duration to investigate these questions.

Additionally, it is acknowledged that internal cognitive variability may account for some of the performance variance observed within these clusters. Future studies may integrate individual differences in cognitive abilities through psychometric baseline testing (e.g., the OSPAN or stroop tests) to further refine these results.

Another direction to explore is to design performance evaluation studies for additional types of populations (e.g., young children, older adults), tasks (with temporal/productivity constraints), task types (co-operative/competitive) and H-R collaborative scenarios (with different coordination protocols; with varied levels of team-coordination proficiency in human and robots; and, with robots programmed with advanced machine learning models) to further understand if the outcome of this study is population-dependent or task-dependent or H-R collaborative scenario dependent.

## Data Availability

The raw data supporting the conclusions of this article will be made available by the authors, without undue reservation.

## References

[ref1] Adamides G. Katsanos C. Parmet Y. Christou G. Xenos M. Hadzilacos T. . (2017). HRI usability evaluation of interaction modes for a teleoperated agricultural robotic sprayer. Appl. Ergon. 62, 237–246. doi: 10.1016/j.apergo.2017.03.008, 28411734

[ref2] Adams J. A. (2009). Multiple robot/single human interaction: effects on perceived workload. Behav. Inf. Technol. 28, 183–198. doi: 10.1080/01449290701288791

[ref3] Almajid R. Keshner E. (2019). Role of gender in dual-tasking timed up and go tests: a cross-sectional study. J. Mot. Behav. 51, 681–689. doi: 10.1080/00222895.2019.1565528, 30676272

[ref4] Anzalone S. M. Boucenna S. Ivaldi S. Chetouani M. (2015). Evaluating the engagement with social robots. Int. J. Soc. Robot. 7, 465–478. doi: 10.1007/s12369-015-0298-7

[ref5] Baltrusch S. J. Krause F. de Vries A. W. van Dijk W. de Looze M. P. (2022). What about the human in human robot collaboration?: a literature review on HRC’s effects on aspects of job quality. Ergonomics 65, 719–740. doi: 10.1080/00140139.2021.1984585 34546152

[ref6] Batsakes P. J. Fisk A. D. (2000). Age-related differences in dual-task visual search: are performance gains retained? J. Gerontol. B 55, P332–P342. doi: 10.1093/geronb/55.6.P332, 11078103

[ref7] Bausenhart K. M. Rolke B. Seibold V. C. Ulrich R. (2010). Temporal preparation influences the dynamics of information processing: evidence for early onset of information accumulation. Vis. Res. 50, 1025–1034. doi: 10.1016/j.visres.2010.03.011, 20338190

[ref8] Bear J. B. Woolley A. W. (2011). The role of gender in team collaboration and performance. Interdiscip. Sci. Rev. 36, 146–153. doi: 10.1179/030801811X13013181961473

[ref9] Bilal D. Kirby J. (2001). Factors influencing children’s and adults’ information seeking on the web: results of two studies. Proceedings of the American Society for Information Science and Technology. 126–140.

[ref10] Bilal D. Kirby J. (2002). Differences and similarities in information seeking: children and adults as web users. Inf. Process. Manag. 38, 649–670. doi: 10.1016/S0306-4573(01)00057-7

[ref11] Biswas M. Murray J. (2016). The effects of cognitive biases and imperfectness in long-term robot-human interactions: case studies using five cognitive biases on three robots. Cogn. Syst. Res. 43, 266–290. doi: 10.1016/j.cogsys.2016.07.007

[ref12] Broadbent E. Stafford R. MacDonald B. (2009). Acceptance of healthcare robots for the older population: review and future directions. Int. J. Soc. Robot. 1, 319–330. doi: 10.1007/s12369-009-0030-6

[ref13] Bukchin J. Luquer R. Shtub A. (2002). Learning in tele-operations. IIE Trans. 34, 245–252. doi: 10.1080/07408170208928866

[ref14] Busch T. (1995). Gender differences in self-efficacy and attitudes toward computers. J. Educ. Comput. Res. 12:147. doi: 10.2190/H7E1-XMM7-GU9B-3HWR

[ref15] Carpenter J. Davis J. M. Erwin-Stewart N. Lee T. R. Bransford J. D. Vye N. (2009). Gender representation and humanoid robots designed for domestic use. Int. J. Soc. Robot. 1, 261–265. doi: 10.1007/s12369-009-0016-4

[ref16] Chandra V. Nehra D. Parent R. Woo R. Reyes R. Hernandez-Boussard T. . (2010). A comparison of laparoscopic and robotic assisted suturing performance by experts and novices. Surgery 147, 830–839. doi: 10.1016/j.surg.2009.11.002, 20045162

[ref17] Charalambous G. Fletcher S. R. Webb P. (2017). The development of a human factors readiness level tool for implementing industrial human-robot collaboration. Int. J. Adv. Manuf. Technol. 91, 2465–2475. doi: 10.1007/s00170-016-9876-6

[ref18] Choi M. Kim S. Park H. (2018). Empirical study on the factors influencing process innovation when adopting intelligent robots at small- and medium-sized enterprises—the role of organizational supports. Information 9:315. doi: 10.3390/info9120315

[ref19] Chuang T. Y. Chen W. F. (2009). Effect of computer-based video games on children: an experimental study. Educ. Technol. Soc. 12, 1–10. doi: 10.1109/DIGITEL.2007.24

[ref20] Chung-En Y. (2018). Humanlike robot and human staff in service: age and gender differences in perceiving smiling behaviors. 7th International Conference on Industrial Technology and Management (ICITM). 99–103

[ref21] Crandall J. W. Goodrich M. a. Olsen D. R. Nielsen C. W. (2005). Validating human–robot interaction schemes in multitasking environments. IEEE Trans. Syst. Man Cybern. A 35, 438–449. doi: 10.1109/TSMCA.2005.850587

[ref22] Eagly A. H. (1987). Sex Differences in Social Behavior: A Social-Role Interpretation. Hillsdale, NJ: Lawrence Erlbaum Associates.

[ref23] Efe B. Kremer G. E. O. Kurt M. (2018). Age and gender based workload constraint for assembly line worker assignment and balancing problem in a textile firm. Int. J. Ind. Eng. Theory Appl. Pract. 25:2961. doi: 10.23055/ijietap.2018.25.1.2961

[ref24] Eizicovits D. Edan Y. Tabak I. Levy-Tzedek S. (2018). Robotic gaming prototype for upper limb exercise: effects of age and embodiment on user preferences and movement. Restor. Neurol. Neurosci. 36, 261–274. doi: 10.3233/RNN-170802, 29526862 PMC5870005

[ref25] Enayati N. Ferrigno G. de Momi E. (2017). Skill-based human–robot cooperation in tele-operated path tracking. Auton. Robot. 42, 997–1009. doi: 10.1007/s10514-017-9675-4

[ref26] Epling S. L. Edgar G. K. Russell P. N. Helton W. S. (2018). How does physical demand affect cognitive performance? interference between outdoor running and narrative memory. Proceedings of the Human Factors and Ergonomics Society Annual Meeting

[ref27] Epling S. L. Edgar G. K. Russell P. N. Helton W. S. (2019). Is semantic vigilance impaired by narrative memory demands? Theory and applications. Hum. Factors 61, 451–461. doi: 10.1177/0018720818805602, 30325648

[ref28] Erikson E. H. (1963). Childhood and Society. New York: W. W. Norton & Company.

[ref29] Farmer G. D. Janssen C. P. Nguyen A. T. Brumby D. P. (2018). Dividing attention between tasks: testing whether explicit payoff functions elicit optimal dual-task performance. Cogn. Sci. 42, 820–849. doi: 10.1111/cogs.12513, 28653447 PMC5969112

[ref30] Feingold-Polak R. Elishay A. Shahar Y. Stein M. Edan Y. Levy-Tzedek S. (2018). Differences between young and old users when interacting with a humanoid robot: a qualitative usability study. Paladyn J. Behav. Robot. 9, 183–192. doi: 10.1515/pjbr-2018-0013

[ref31] Fong T. Scholtz J. Shah J. A. Fluckiger L. Kunz C. Lees D. . (2006). A preliminary study of peer-to-peer human-robot interaction. IEEE International Conference on Systems, Man and Cybernetics. 3198–3203

[ref32] Garbie I. H. (2014). An experimental investigation on ergonomically designed assembly workstation. Int. J. Ind. Syst. Eng. 16:296. doi: 10.1504/IJISE.2014.060131

[ref33] Gefen D. Straub D. W. (1997). Gender differences in the perception and use of E-mail: an extension to the technology acceptance model. MIS Q. 21, 389–400. doi: 10.2307/249720

[ref34] Ghazali A. S. Ham J. Barakova E. I. Markopoulos P. (2018). Effects of robot facial characteristics and gender in persuasive human-robot interaction. Front. Robot. AI 5:73. doi: 10.3389/frobt.2018.00073, 33500952 PMC7805818

[ref35] Goddard L. Dritschel B. Burton A. (1998). Gender differences in the dual-task effects on autobiographical memory retrieval during social problem solving. Br. J. Psychol. 89, 611–627. doi: 10.1111/j.2044-8295.1998.tb02706.x, 9854806

[ref36] Gong G. Deng Q. Gong X. Liu W. Ren Q. (2018). A new double flexible job-shop scheduling problem integrating processing time, green production, and human factor indicators. J. Clean. Prod. 174, 560–576. doi: 10.1016/j.jclepro.2017.10.188

[ref37] Gutman D. Olatunji S. Edan Y. (2021). Evaluating levels of automation in human–robot collaboration at different workload levels. Appl. Sci. 11:7340. doi: 10.3390/app11167340

[ref38] Heerink M. (2011). Exploring the influence of age, gender, education and computer experience on robot acceptance by older adults. 6th ACM/IEEE International Conference on Human-Robot Interaction (HRI). 147–148

[ref39] Hoffman G. (2013). Evaluating fluency in human-robot collaboration. Robotics Science and Systems (RSS), Workshop on Human Robot Collaboration.

[ref40] Hoffman G. (2019). Evaluating fluency in human–robot collaboration. IEEE Trans. Hum. Mach. Syst. 49, 209–218. doi: 10.1109/THMS.2019.2904558

[ref41] Hollman J. H. Youdas J. W. Lanzino D. J. (2011). Gender differences in dual task gait performance in older adults. Am. J. Mens Health 5, 11–17. doi: 10.1177/1557988309357232, 20031935

[ref42] Hourcade J. P. Bederson B. B. Druin A. Guimbretière F. (2004). Differences in pointing task performance between preschool children and adults using mice. ACM Trans. Comput. Hum. Interact. 11, 357–386. doi: 10.1145/1035575.1035577

[ref43] Howard A. (2005). A methodology to assess performance of human-robotic systems in achievement of collective tasks. IEEE/RSJ International Conference on Intelligent Robots and Systems. 90–95.

[ref44] Howard A. M. (2007). A systematic approach to predict performance of human–automation systems. IEEE Trans. Syst. Man Cybern. C 37, 594–601. doi: 10.1109/tsmcc.2007.897505

[ref45] Huang C.-M. Cakmak M. Mutlu B. (2015). Adaptive coordination strategies for human-robot handovers. 2015 Robotics: Science and Systems Conference

[ref46] Jenkins J. L. Anderson B. B. Vance A. Kirwan C. B. Eargle D. (2016). More harm than good? How messages that interrupt can make us vulnerable. Inf. Syst. Res. 27, 665–991. doi: 10.1287/isre.2016.0644

[ref47] Judkins T. N. Oleynikov D. Stergiou N. (2009). Objective evaluation of expert and novice performance during robotic surgical training tasks. Surg. Endosc. 23, 590–597. doi: 10.1007/s00464-008-9933-9, 18443870

[ref48] Kang N. E. Yoon W. C. (2008). Age- and experience-related user behavior differences in the use of complicated electronic devices. Int. J. Hum. Comput. Stud. 66, 425–437. doi: 10.1016/j.ijhcs.2007.12.003

[ref49] Kaplan K. E. Nichols K. A. Okamura A. M. (2016). Toward human-robot collaboration in surgery: performance assessment of human and robotic agents in an inclusion segmentation task. 2016 IEEE International Conference on Robotics and Automation (ICRA). 723–729

[ref50] Katiraee N. Battini D. Battaia O. Calzavara M. (2019). Human diversity factors in production system modelling and design: state of the art and future researches. IFAC-PapersOnLine 52, 2544–2549. doi: 10.1016/j.ifacol.2019.11.589

[ref51] Khan Z. (1998). “Attitude towards intelligent service robots numerical analysis and computer science,” in Technical Report (TRITA-NA-P9821), (Stockholm: Royal Institute of Technology).

[ref52] Kim W. Y. Ko S. Y. Park J. O. Park S. (2015). 6-DOF force feedback control of robot-assisted bone fracture reduction system using double F/T sensors and adjustable admittances to protect bones against damage. Mechatronics 35, 136–147. doi: 10.1016/j.mechatronics.2016.02.005

[ref53] Kimura T. Matsuura R. (2020). Additional effects of a cognitive task on dual-task training to reduce dual-task interference. Psychol. Sport Exerc. 46:101588. doi: 10.1016/j.psychsport.2019.101588

[ref54] Korteling J. (1991). Effects of skill integration and perceptual competition on age-related differences in dual-task performance. Hum. Factors 33, 35–44. doi: 10.1177/001872089103300103, 2037307

[ref55] Korteling J. (1993). Effects of age and task similarity on dual-task performance. Hum. Factors 35, 99–113. doi: 10.1177/001872089303500106, 8509106

[ref56] Korteling J. (1994). Effects of aging, skill modification, and demand alternation on multiple- task performance. Hum. Factors 36, 27–43. doi: 10.1177/001872089403600102, 8026841

[ref57] Kovess-Masfety V. Keyes K. Hamilton A. Hanson G. Bitfoi A. Golitz D. . (2016). Is time spent playing video games associated with mental health, cognitive and social skills in young children? Soc. Psychiatry Psychiatr. Epidemiol. 51, 349–357. doi: 10.1007/s00127-016-1179-6, 26846228 PMC4814321

[ref58] Kramer A. F. Larish J. F. (1996). “Aging and dual-task performance,” in Aging and Skilled Performance, (Hillsdale, NJ: Lawrence Erlbaum Associates).

[ref59] Kuchenbrandt D. Häring M. Eichberg J. Eyssel F. André E. (2014). Keep an eye on the task! How gender typicality of tasks influence human-robot interactions. Int. J. Soc. Robot. 6, 417–427. doi: 10.1007/s12369-014-0244-0

[ref60] Kuo I. H. Rabindran J. M. Broadbent E. Lee Y. I. Kerse N. Stafford R. M. Q. . (2009). Age and gender factors in user acceptance of healthcare robots. 18th IEEE International Symposium on Robot and Human Interactive Communication. 214–219

[ref61] Labonte D. Boissy P. Michaud F. (2010). Comparative analysis of 3-D robot teleoperation interfaces with novice users. IEEE Trans. Syst. Man Cybern. B 40, 1331–1342. doi: 10.1109/TSMCB.2009.2038357, 20106745

[ref62] Lasota P. A. Shah J. A. (2015). Analyzing the effects of human-aware motion planning on close-proximity human-robot collaboration. Hum. Factors 57, 21–33. doi: 10.1177/0018720814565188, 25790568 PMC4359211

[ref63] Liepelt R. Strobach T. Frensch P. Schubert T. (2011). Improved intertask coordination after extensive dual-task practice. Q. J. Exp. Psychol. 64, 1251–1272. doi: 10.1080/17470218.2010.543284, 21462091

[ref64] Lin C. H. Liu E. Z. F. Huang Y. Y. (2012). Exploring parents’ perceptions towards educational robots: gender and socio-economic differences. Br. J. Educ. Technol. 43, E31–E34. doi: 10.1111/j.1467-8535.2011.01258.x

[ref65] Liu L. Guo F. Zou Z. Duffy V. G. (2022). Application, development and future opportunities of collaborative robots (cobots) in manufacturing: a literature review. Int. J. Hum. Comput. Interact. 40, 915–932. doi: 10.1080/10447318.2022.2041907

[ref66] Liu M. Yang X. (2019). Bi-objective optimization for scheduling and multi-skilled worker assignments in the hybrid flow shop. IFAC-PapersOnLine 52, 2128–2133. doi: 10.1016/j.ifacol.2019.11.520

[ref67] Lohani M. Stokes C. Dashan N. McCoy M. Bailey C. A. Rivers S. E. (2017). “A framework for human-agent social systems: the role of non-technical factors in operation success,” in Advances in Human Factors in Robots and Unmanned Systems. Advances in Intelligent Systems and Computing, eds. Savage-Knepshield P. Chen J. (Cham: Springer), 137–148.

[ref68] Lorsbach T. C. Simpson G. B. (1988). Dual-task performance as a function of adult age and task complexity. Psychol. Aging 3, 210–212. doi: 10.1037/0882-7974.3.2.210, 3268261

[ref69] Margrett J. A. Marsiske M. (2002). Gender differences in older adults’ everyday cognitive collaboration. Int. J. Behav. Dev. 26, 45–59. doi: 10.1080/01650250143000319, 20657668 PMC2909137

[ref70] Maroto-Gómez M. Castro-González Á. Castillo J. C. Malfaz M. Salichs M. Á. (2022). An adaptive decision-making system supported on user preference predictions for human–robot interactive communication. User Model. User Adap. Inter. 33, 359–403. doi: 10.1007/s11257-022-09321-2, 35431456 PMC8994572

[ref71] Maurice P. Padois V. Measson Y. Bidaud P. (2017). Human-oriented design of collaborative robots. Int. J. Ind. Ergon. 57, 88–102. doi: 10.1016/j.ergon.2016.11.011

[ref72] McDowd J. M. (1986). The effects of age and extended practice on divided attention performance. J. Gerontol. 41, 764–769. doi: 10.1093/geronj/41.6.764, 3772053

[ref73] McDowd J. M. Craik F. I. M. (1988). Effects of aging and task difficulty on divided attention performance. J. Exp. Psychol. Hum. Percept. Perform. 14, 267–280. doi: 10.1037/0096-1523.14.2.267, 2967880

[ref74] McDowd J. M. Vercruyssen M. Birren J. E. (1991). “Aging, divided attention, and dual-task performance,” in Multiple Task Performance, (Boca Raton, FL: CRC Press), 387–414.

[ref75] McGinn C. Sena A. Kelly K. (2017). Controlling robots in the home: factors that affect the performance of novice robot operators. Appl. Ergon. 65, 23–32. doi: 10.1016/j.apergo.2017.05.005, 28802443

[ref76] Meyer Zu Borgsen S. Bernotat J. Wachsmuth S. (2017). Hand in hand with robots: differences between experienced and naive users in human-robot handover scenarios. International Conference on Social Robotics. 587–596

[ref77] Michaelis J. E. Siebert-Evenstone A. Shaffer D. W. Mutlu B. (2020). Collaborative or simply uncaged? Understanding human-cobot interactions in automation. Proceedings of the Conference on Human Factors in Computing Systems

[ref78] Milliez G. Lallement R. Fiore M. Alami R. (2016). “Using human knowledge awareness to adapt collaborative plan generation, explanation and monitoring,” in 2016 11th ACM/IEEE International Conference on Human-Robot Interaction (HRI), (43–50.

[ref79] Moniz A. Krings B.-J. (2016). Robots working with humans or humans working with robots? Searching for social dimensions in new human-robot interaction in industry. Societies 6:23. doi: 10.3390/soc6030023

[ref80] Morris M. G. Venkatesh V. (2000). Age differences in technology adoption decisions: implications for a changing work force. Pers. Psychol. 53, 375–403. doi: 10.1111/j.1744-6570.2000.tb00206.x

[ref81] Muhs K. S. Karwowski W. Kern D. (2018). Temporal variability in human performance: a systematic literature review. Int. J. Ind. Ergon. 64, 31–50. doi: 10.1016/j.ergon.2017.10.002

[ref82] Mutlu B. Osman S. Forlizzi J. Hodgins J. Kiesler S. (2006). Task structure and user attributes as elements of human-robot interaction design. 15th IEEE International Symposium on Robot and Human Interactive Communication. 74–79

[ref83] Niculescu A. Hofs D. van Dijk B. Nijholt A. (2010). How the agent's gender influence users’ evaluation of a QA system. 2010 International Conference on User Science and Engineering (i-USEr)

[ref84] Nikolaidis S. Hsu D. Srinivasa S. (2017a). Human-robot mutual adaptation in collaborative tasks: models and experiments. Int. J. Robot. Res. 36, 618–634. doi: 10.1177/0278364917690593, 32855581 PMC7449140

[ref85] Nikolaidis S. Lasota P. Ramakrishnan R. Shah J. (2015). Improved human-robot team performance through cross-training, an approach inspired by human team training practices. Int. J. Robot. Res. 34, 1711–1730. doi: 10.1177/0278364915609673

[ref86] Nikolaidis S. Nath S. Procaccia A. D. Srinivasa S. (2017b). Game-theoretic modeling of human adaptation in human-robot collaboration. Proceedings of the 2017 ACM/IEEE International Conference on Human-Robot Interaction. 323–331

[ref87] Nomura T. (2017). “Robots and gender,” in Principles of Gender-Specific Medicine, (Amsterdam: Elsevier), 695–703.

[ref88] Nomura T. Nakao A. (2010). Comparison on identification of affective body motions by robots between elder people and university students: a case study in Japan. Int. J. Soc. Robot. 2, 147–157. doi: 10.1007/s12369-010-0050-2

[ref89] Oliff H. Liu Y. Kumar M. Williams M. (2020). Improving human-robot interaction utilizing learning and intelligence: a human factors-based approach. IEEE Trans. Autom. Sci. Eng. 17, 1597–1610. doi: 10.1109/TASE.2020.2967093

[ref90] Ong C. S. Lai J. Y. (2006). Gender differences in perceptions and relationships among dominants of e-learning acceptance. Comput. Human Behav. 22, 816–829. doi: 10.1016/j.chb.2004.03.006

[ref91] Parasuraman R. Galster S. Squire P. Furukawa H. Miller C. (2005). A flexible delegation-type interface enhances system performance in human supervision of multiple robots: empirical studies with RoboFlag. IEEE Trans. Syst. Man Cybern. Syst. Hum. 35, 481–493. doi: 10.1109/TSMCA.2005.850598

[ref2000] Pashler H. (1994). Dual-task interference in simple tasks: data and theory. Psychological bulletin. 116:220. 10.1037/0033-2909.116.2.220 7972591

[ref92] Pearce M. Mutlu B. Shah J. Radwin R. (2018). Optimizing makespan and ergonomics in integrating collaborative robots into manufacturing processes. IEEE Trans. Autom. Sci. Eng. 15, 1772–1784. doi: 10.1109/TASE.2018.2789820

[ref93] Pilcher J. J. Jennings K. S. Phillips G. E. McCubbin J. A. (2016). Auditory attention and comprehension during a simulated night shift: effects of task characteristics. Hum. Factors 58, 1031–1043. doi: 10.1177/0018720816654377, 27307380

[ref94] Pizoń J. Gola A. (2023). Human-machine relationship: perspective and future roadmap for industry 5.0 solutions. Machines 11:203. doi: 10.3390/MACHINES11020203

[ref95] Prewett M. S. Johnson R. C. Saboe K. N. Elliott L. R. Coovert M. D. (2010). Managing workload in human-robot interaction: a review of empirical studies. Comput. Hum. Behav. 26, 840–856. doi: 10.1016/j.chb.2010.03.010

[ref96] Rabbani M. Akbari E. Dolatkhah M. (2017). Manpower allocation in a cellular manufacturing system considering the impact of learning, training and combination of learning and training in operator skills. Manage. Sci. Lett. 7, 9–22. doi: 10.5267/j.msl.2016.11.006

[ref97] Rahman S. M. M. (2018). Cyber-physical-social system between a humanoid robot and a virtual human through a shared platform for adaptive agent ecology. IEEE/CAA J. Autom. Sinica 5, 190–203. doi: 10.1109/JAS.2017.7510760

[ref98] Realmuto J. Warrier R. B. Devasia S. (2017). Data-inferred personalized human-robot models for iterative collaborative output tracking. J. Intell. Robot. Syst. 91, 137–153. doi: 10.1007/s10846-017-0653-z

[ref99] Reinerman-Jones L. Barber D. J. Szalma J. L. Hancock P. A. (2017). Human interaction with robotic systems: performance and workload evaluations. Ergonomics 60, 1351–1368. doi: 10.1080/00140139.2016.1254282, 28745552

[ref100] Rill R. A. Faragó K. B. Lőrincz A. (2018). Strategic predictors of performance in a divided attention task. PLoS One 13:e0195131. doi: 10.1371/journal.pone.0195131, 29621292 PMC5886418

[ref101] Rogers R. D. Monsell S. (1995). Costs of a predictable switch between simple cognitive tasks. J. Exp. Psychol. Gen. 124, 207–231. doi: 10.1037/0096-3445.124.2.207

[ref102] Rossi S. Ferland F. Tapus A. (2017). User profiling and behavioral adaptation for HRI: a survey. Pattern Recogn. Lett. 99, 3–12. doi: 10.1016/j.patrec.2017.06.002

[ref103] Roy S. (2016). “Designing robots as teammates: human-robot collaboration models for temporal coordination in human-robot teams,” in Ph.D. Thesis, (Be’er Sheva: Ben-Gurion University of the Negev).

[ref104] Roy S. Edan Y. (2017). Givers & receivers perceive handover tasks differently: implications for human-robot collaborative system design. *arXiv*. Available online at: 10.48550/arXiv.1708.06207. [Epub ahead of preprint]

[ref105] Roy S. Edan Y. (2020). Investigating joint-action in short-cycle repetitive handover tasks: the role of giver versus receiver and its implications for human-robot collaborative system design. Int. J. Soc. Robot. 12, 973–988. doi: 10.1007/s12369-017-0424-9

[ref106] Sadeghi P. Rebelo R. D. Ferreira J. S. (2018). Balancing mixed-model assembly systems in the footwear industry with a variable neighbourhood descent method. Comput. Ind. Eng. 121, 161–176. doi: 10.1016/j.cie.2018.05.020

[ref107] Salthouse T. A. Rogan J. D. Prill K. A. (1984). Division of attention: age differences on a visually presented memory task. Mem. Cogn. 12, 613–620. doi: 10.3758/BF03213350, 6533430

[ref108] Sanabria D. Capizzi M. Correa A. (2011). Rhythms that speed you up. J. Exp. Psychol. Hum. Percept. Perform. 37, 236–244. doi: 10.1037/a0019956, 20718571

[ref109] Sandygulova A. O’Hare G. M. P. (2018). Age- and gender-based differences in children’s interactions with a gender-matching robot. Int. J. Soc. Robot. 10, 687–700. doi: 10.1007/s12369-018-0472-9

[ref110] Sanneman L. Fourie C. Shah J. A. (2021). The state of industrial robotics: emerging technologies, challenges, and key research directions. Robotics 8, 225–306. doi: 10.1561/2300000065

[ref111] Sarkar N. (2002). Psychophysiological control architecture for human-robot coordination-concepts and initial experiments. Proceedings 2002 IEEE International Conference on Robotics and Automation. 3719–3724

[ref112] Sayfeld L. Peretz Y. Someshwar R. Edan Y. (2015). Evaluation of human-robot collaboration models for fluent operations in industrial tasks. Robotics: Science and Systems Conference (RSS), Human-Robot Hand-Over Workshop

[ref113] Schrepp M. Hinderks A. Thomaschewski J. (2017). Design and evaluation of a short version of the User Experience Questionnaire (UEQ-S). Int. J. Interact. Multimed. Artif. Intell. 4, 103–108. doi: 10.9781/ijimai.2017.09.001

[ref114] Scopelliti M. Giuliani M. V. Fornara F. (2005). Robots in a domestic setting: a psychological approach. Universal Access Inf. Soc. 4, 146–155. doi: 10.1007/s10209-005-0118-1

[ref115] Shanteau J. Weiss D. J. Thomas R. P. Pounds J. C. (2002). Performance-based assessment of expertise: how to decide if someone is an expert or not. Eur. J. Oper. Res. 136, 253–263. doi: 10.1016/S0377-2217(01)00113-8

[ref116] Sheridan T. B. (2020). A review of recent research in social robotics. Curr. Opin. Psychol. 36, 7–12. doi: 10.1016/j.copsyc.2020.01.003, 32294577

[ref117] Simões A. C. Pinto A. Santos J. Pinheiro S. Romero D. (2022). Designing human-robot collaboration (HRC) workspaces in industrial settings: a systematic literature review. J. Manuf. Syst. 62, 28–43. doi: 10.1016/j.jmsy.2021.11.007

[ref118] Singer S. Akin D. (2011). A survey of quantitative team performance metrics for human-robot collaboration. 41st International Conference on Environmental Systems. 1–19

[ref119] Sit R. A. Fisk A. D. (1999). Age-related performance in a multiple-task environment. Hum. Factors 41, 26–34. doi: 10.1518/001872099779577345, 10354804

[ref120] Skantze G. (2017). Predicting and regulating participation equality in human-robot conversations. Proceedings of the 2017 ACM/IEEE International Conference on Human-Robot Interaction. 196–204

[ref121] Somberg B. L. Salthouse T. A. (1982). Divided attention abilities in young and old adults. J. Exp. Psychol. Hum. Percept. Perform. 8, 651–663. doi: 10.1037/0096-1523.8.5.651, 6218227

[ref122] Someshwar R. Kerner Y. (2013). Optimization of waiting time in H-R coordination. IEEE International Conference on Systems, Man, and Cybernetics (SMC). 1918–1923

[ref123] Someshwar R. Meyer J. Edan Y. (2012). A timing control model for H-R synchronization. IFAC Proc. Vol. 45, 698–703. doi: 10.3182/20120905-3-HR-2030.00134

[ref124] Strayer D. L. Johnston W. A. (2001). Driven to distraction: dual-task studies of simulated driving and conversing on a cellular telephone. Psychol. Sci. 12, 462–466. doi: 10.1111/1467-9280.00386, 11760132

[ref125] Strobach T. Frensch P. A. Soutschek A. Schubert T. (2012). Investigation on the improvement and transfer of dual-task coordination skills. Psychol. Res. 76, 794–811. doi: 10.1007/s00426-011-0381-0, 21947746

[ref126] Tapiro H. Oron-Gilad T. Parmet Y. (2018). The effect of environmental distractions on child pedestrian’s crossing behavior. Saf. Sci. 106, 219–229. doi: 10.1016/j.ssci.2018.03.024

[ref127] Unhelkar V. V. Lasota P. A. Tyroller Q. Buhai R.-D. Marceau L. Deml B. . (2018). Human-aware robotic assistant for collaborative assembly: integrating human motion prediction with planning in time. IEEE Rob. Autom. Lett. 3, 2394–2401. doi: 10.1109/LRA.2018.2812906

[ref128] Verhaeghen P. Steitz D. W. Sliwinski M. J. Cerella J. (2003). Aging and dual-task performance: a meta-analysis. Psychol. Aging 18, 443–460. doi: 10.1037/0882-7974.18.3.443 14518807

[ref129] Wang D. Proctor R. W. Pick D. F. (2007). Acquisition and transfer of attention allocation strategies in a multiple-task work environment. Hum. Factors 49, 995–1004. doi: 10.1518/001872007X249866 18074699

[ref130] Wang Y. Wu M. C. Wang H. Y. (2009). Investigating the determinants and age and gender differences in the acceptance of mobile learning. Br. J. Educ. Technol. 40, 92–118. doi: 10.1111/j.1467-8535.2007.00809.x

[ref131] Wolf T. Sebanz N. Knoblich G. (2018). Joint action coordination in expert-novice pairs: can experts predict novices’ suboptimal timing? Cognition 178, 103–108. doi: 10.1016/j.cognition.2018.05.012, 29852424 PMC6039761

[ref132] Wolff P. H. Cohen C. (1980). Dual task performance during bimanual coordination. Cortex 16, 119–133. doi: 10.1016/S0010-9452(80)80027-X 7379557

[ref133] Wong C. Y. Seet G. (2017). Workload, awareness and automation in multiple-robot supervision. Int. J. Adv. Robot. Syst. 14. doi: 10.1177/1729881417710463

[ref134] Woodham A. Billinghurst M. Helton W. S. (2016). Climbing with a head-mounted display: dual-task costs. Hum. Factors 58, 452–461. doi: 10.1177/0018720815623431, 26865416

[ref135] Wright J. L. Chen J. Y. C. Barnes M. J. (2018). Human–automation interaction for multiple robot control: the effect of varying automation assistance and individual differences on operator performance. Ergonomics 61, 1033–1045. doi: 10.1080/00140139.2018.1441449, 29451105

[ref136] Xin B. Li Y. Yu J. Zhang J. (2015). An adaptive BPSO algorithm for multi-skilled workers assignment problem in aircraft assembly lines. Assem. Autom. 35, 317–328. doi: 10.1108/AA-06-2015-051

[ref137] Yeung N. Monsell S. (2003). Switching between tasks of unequal familiarity: the role of stimulus-attribute and response-set selection. J. Exp. Psychol. Hum. Percept. Perform. 29, 455–469. doi: 10.1037/0096-1523.29.2.455, 12760628

